# New Implications on Genomic Adaptation Derived from the *Helicobacter pylori* Genome Comparison

**DOI:** 10.1371/journal.pone.0017300

**Published:** 2011-02-28

**Authors:** Edgar Eduardo Lara-Ramírez, Aldo Segura-Cabrera, Xianwu Guo, Gongxin Yu, Carlos Armando García-Pérez, Mario A. Rodríguez-Pérez

**Affiliations:** 1 Centro de Biotecnología Genómica, Instituto Politécnico Nacional, Reynosa, México; 2 Department of Biological Sciences, Boise State University, Boise, Idaho, United States of America; 3 Department of Informatics and Automatics, Facultad de Ciencias, Universidad de Salamanca, Salamanca, España; University of Hyderabad, India

## Abstract

**Background:**

*Helicobacter pylori* has a reduced genome and lives in a tough environment for long-term persistence. It evolved with its particular characteristics for biological adaptation. Because several *H. pylori* genome sequences are available, comparative analysis could help to better understand genomic adaptation of this particular bacterium.

**Principal Findings:**

We analyzed nine *H. pylori* genomes with emphasis on microevolution from a different perspective. Inversion was an important factor to shape the genome structure. Illegitimate recombination not only led to genomic inversion but also inverted fragment duplication, both of which contributed to the creation of new genes and gene family, and further, homological recombination contributed to events of inversion. Based on the information of genomic rearrangement, the first genome scaffold structure of *H. pylori* last common ancestor was produced. The core genome consists of 1186 genes, of which 22 genes could particularly adapt to human stomach niche. *H. pylori* contains high proportion of pseudogenes whose genesis was principally caused by homopolynucleotide (HPN) mutations. Such mutations are reversible and facilitate the control of gene expression through the change of DNA structure. The reversible mutations and a quasi-panmictic feature could allow such genes or gene fragments frequently transferred within or between populations. Hence, pseudogenes could be a reservoir of adaptation materials and the HPN mutations could be favorable to *H. pylori* adaptation, leading to HPN accumulation on the genomes, which corresponds to a special feature of *Helicobacter* species: extremely high HPN composition of genome.

**Conclusion:**

Our research demonstrated that both genome content and structure of *H. pylori* have been highly adapted to its particular life style.

## Introduction

*Helicobacter pylori* is a human gastric pathogen that infected approximately 50% of the human population. The majority of infected people are asymptomatic but up to 20% of them developed severe diseases, such as peptic ulcer, gastric adenocarcinoma, and MALT (mucous-associated lymphoid tissue)-lymphoma [1]. It is the first bacterium that was identified as class I carcinogen by WHO in 1994. *H. pylori* host are limited to human or primates, without other natural reservoir [2], [3]. When *H. pylori* infected human, it can persist for decades. The *H. pylori* diversity researches on the human population sampling from Asia, Africa, and South America demonstrated that *H. pylori*-human co-evolution has been for about 58,000 years [4], [5], [6], [7].

This bacterium has been developed to highly adapt to human stomach on the way of the coevolution. The human stomach is a harsh environment to bacteria due to very low pH and many enzymes in the mucosa for digestion. *H. pylori* synthesizes urease to neutralize the acidic environment surrounding the bacterium by converting urea to ammonia and carbon dioxide [8]. This bacterium is natural competence cell and developed a specified Type IV Secretion System (T4SS), the *comB*-system, to integrate exogenous DNA into its genome through genetic recombination [9], [10]. Human stomach has low bacterial diversity on the level of species but is rich in genetic variants in subpopulations of *H. pylori*. The maintenance of high diversification makes this bacterium to cope with particular challenges in individual hosts [11].

In the process of adaptation, bacteria need to accumulate sufficient mutations for challenging new niches. In addition to point mutations, other mutations were generally shown as the changes in genomic structure: inversion, transposition, translocation and duplication, gene gain and gene lost, gene fusion and gene split, gene fragmentation (pseudogene) or insertion and deletion (indel). In *H. pylori*, genomic inversion was common. One feature of the inversion is replication-directed, leading to being symmetric around the replication axis [12], [13], [14]. Direct repeats had also important role in *H. pylori* DNA diversification [15]. Contingency genes expressed as in on-off switch in progression of phase variation by the change of DNA structure [16], [17], [18], [19], [20], [21], [22]. *H. pylori* has high rate of mutation and recombination that allow a quasi-panmictic population for rapid adaptation to a new environment [23], [24].

*H. pylori* has a reduced genome, only from 1.5 to 1.7 Mb. At the time of this writing, nine *H. pylori* genome sequences are available from public databases: 26695 (accession number: AE000511) [25], J99 (AE001439.1)[26], P12 (EMBL:CP001217, EMBL:CP001218 for plasmid]) [27], HPAG1 (CP000241, CP000242 for plasmid) [28]), or G27 (CP001173,CP001174 from plasmid) [29], Shi470 (CP001072) [30], B38 (FM991728)[31], 51(CP000012) and 52 (CP001680). These nine genomes represent the genetic information of isolates from patients with various diseases (from gastritis to cancer) from different geographic regions. Although several articles on the comparisons of *H. pylori* genomes have been published [26], [27], [28], [30], [31], [32], [33], in the present paper, we compared nine genomes with emphasis on microevolution in a different perspective, which will help to understand the nature of adaptation of this special bacterium.

## Materials and Methods

### Whole genome multi-alignments

All the genome sequences were downloaded from NCBI database. Whole genome alignments of the nine *H. pylori* test strains were performed using Mauve version 2.3.1. with the default MauveAligner parameters [34]. This method utilizes pairwise or multiple alignments of conserved sequences for whole genomes, with modest computational requirements without compromising the alignment quality. Local alignments were performed to identify multiple maximal unique matches (multi-MUMs), which were subsequently used to calculate a guide tree construction. Subsets of multi-MUMs were then used as anchors, and were divided into local collinear blocks. Each block is a homologous DNA region of multi-MUMs, which lacks any sequence rearrangements and is shared by two or more genomes under analysis. The sequence alignment allows to identify the number of common collinear blocks by using the length of total conserved regions and the overall nucleotide identity between chromosomal sequences for each pairs of strains. In addition, we aligned the nine *H. pylori* genomes with MUMmer 3.0 software [35].

### DNA repeat analysis

REPuter [36] was used to categorize the DNA sequence repeats within the genomes of the nine genomes of *H.* pylori. This method allows the identification of inverted and direct repeats. We used 25 nucleotides as the minimum cut-off length of the DNA repeat sequences.

### Core genome determination

The proteomes from nine *H. pylori strains*, and *Helicobacter acinonychis* as well were downloaded from GenGank and orthologs were determined using OrthoMCL [37]. This program first maks an all-against-all BLASTp, and then defines putative pairs of orthologs or recent paralogs based on reciprocal BLAST. Recent paralogs are identified as genes within the genome that are reciprocally more similar than any sequences from other genomes. OrthoMCL then converts the reciprocal BLAST *p*-values to a normalized similarity matrix that was analyzed by a Markov Cluster algorithm (MCL) (http://www.micans.org/mcl). MCL yielded a set of clusters, each of which contained a set of orthologs and/or recent paralogs. OrthoMCL was run with a BLAST *E*-value cut-off of 1e^−6^ and an inflation parameter of 1.5. We used the OrthoMCl output to determine genome gene content. The lists generated by OrthoMCL were manually inspected to determine the core genome while the genes that were not included in the OrthoMCl output were considered as strain-specific gene.

### Ancestral genome reconstruction

The 34 local colinear blocks shared among the nine *Helicobacter* genomes, produced by Mauve, were encoded as a signed permutation matrix to indicate order and orientation of homologous segments in a genome. The signed permutation matrix was submitted to the MGR (Multiple Genome Rearrangements) program [38] and run with default parameters to reconstruct the putative ancestral genome order and create the phylogenomic tree based on the history of the inversions.

### Alignments and phylogenetic analysis

The gene sequences analyzed in the present study were aligned with the MUSCLE software [39]. The 40 selected core genes from each genome were used for analysis (Table S1). Multi-alignments with MUSCLE were respectively applied to each individual gene and four groups of each 10 concatenated genes. The alignments files generated were then submitted to the graphical interface of SeaView version 4 software [40] to construct phylogenetic trees using the neighbor-joining method with bootstrap of 1000 replications.

### Premature mutation finding and classification of mutations

The premutations from *H. pylori* strains were determined using program GenVar [41] in combination with the annotation of Genbank. The DNA multi-alignments of pseudogene and its orthologs from different strains were performed firstly. We then distinguished the types of mutations of this pseudogene from the normal sequences of other strains. For the large insertion in a specific strain, the programs Etandem [42] and REPuter [36] were needed to help for analysis.

### Calculation of proportion of homopolynucleotide (HPN) sequences in a genome

We developed a program in our laboratory with Pearl script codes to search for the HPN repeats within the genomes tested in the present study. This program is available from the authors upon request. The proportion of a HPN in a genome was calculated as: the number of a HPN in a genome× the length of the HPN/genome size (bp).

## Results and Discussion

### Inversions and Inverted sequences

The structure of nine genomes has been analyzed with Mauve (J99 as a reference strain). Based on the homologous DNA sequences among genomes, each genome has been divided into 34 colinear blocks. It showed that the genome synteny has been interrupted mainly by inversions except a few deletions and translocations, as shown in strains B38 and 26695 (Figure S1). Some translocations could be actually explained by multiple inversions, as shown in the comparison of gene order between the genomes of strain 52 and the strain 26695. Thus, inversion is one of the major causes for the change of genomic synteny. The plasticity regions were not fixed in a specific region among the genomes, which corresponds to be as a transposable unit [43]. In the cancer strain B38, the *cag* island did not exist [31].

**Figure 1:**
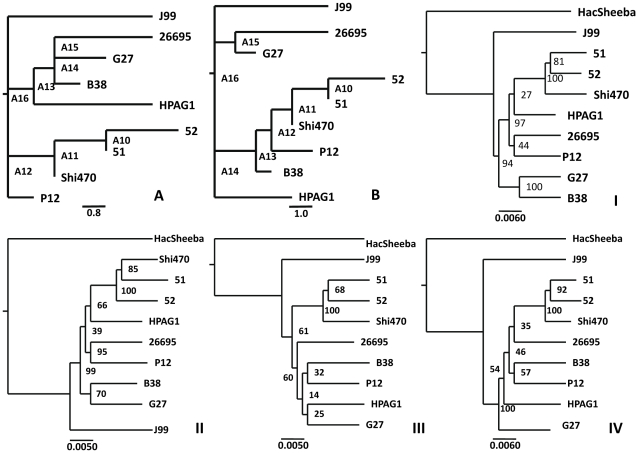
Phylogenic trees of strains based on the genomic rearrangements using different reference genomes (A and B) or on the sequences of 4 groups of each 10 concatenated genes (I–IV). **A**. using reference genome from strains J99, HPAG1, Shi470 or P12. **B**. using reference genome from strains 26695, B38, 51, 52 or G27. A10-A16 represents the ancestor genome structure at different evolutionary stage and A16 is the last common ancient of *H. pylori*. I–IV represents the phylogeny inferred from each group of 10 concatenated core genes.

It is common that many strains share the same inversions, indicating that inversion is an historical event on the way of evolution. The inversion history could thus be the reflection of evolutionary history, from which ancient genome structure of *H. pylori* may be inferred. We used the data from the analysis with Mauve and applied this set of data to the program MGR to produce the scaffold genome structure of last common ancestor [38]. We alternatively applied each genome as a reference to test the influence of distinct reference genome on the analysis. Two forms of phylogenic trees on genome structures were produced: one (form A) using the reference genome from strains J99, HPAG1, SHI470 or P12 and another (form B) using the reference genome from strains 26695, B38, 51, 52 or G27 (Figure 1a, b). The difference between these two phylogenic trees is the relative relationship of some European strains to Asian and Latin-American strains. To evaluate the result, we constructed the phylogeny of nine *H. pylori* strains based on the sequences of each of 40 core genes of *H. pylori* (Table S1). The individual gene phylogenic analysis showed that it was difficult to produce the congruent result, particularly for the Europe and Northern America strains. To overcome the problem, the 40 genes were divided into four groups, each of which had 10 genes. The sequences of 10 concatenated genes were used to produce phylogenic trees, as shown in Figure 1. I, II, III, IV). They were not exactly congruent but it can be concluded according to the plurality rule that: 1. J99 had been separated earlier from the last ancient ancestor; 2. Shi470 was more ancient strain than the Asian strains, 51 and 52 and the three strains formed a group. 3. European or North-American strains contained more ancient genome structure than the Asian Latin-American strains. These results are in accordance to our analysis on genome structure and those documented by the previous researches on the evolution of *H. pylori* [4], [5], [6], [7]. Therefore, the draft structures of *H. pylori* ancient genome produced by our analysis could be acceptable in general sense. We then analyzed the genome structures of last common ancestor (A16) (Figure 1a, b). From nine genomes, MGR gave the same A16 genome structure by the use of strains J99, HPAG1, SHI470 or P12 as reference (Table 1), but produced incongruent A16 structures using other strains as reference, in which one or more gene blocks occupied the same position but in different orientation (inversions). We noted that all the congruent A16 structures came from the phylogenic tree form A, in which the strains J99, HPAG1, SHI470 or P12 showed more ancient genome structure (Figure 1a). This matching made the obtained ancient genome structure of *H. pylori* more reasonable. Therefore, we consider this A16 genome structure as the scaffold of last common ancestor genome of *H. pylori*.

**Table 1:** Ancient genome order of last common ancestor (A16) of *H. pylori*.

A16 order	J99 as reference	A16 order	HPAG1 as reference	A16 order	P12 as reference	A16 order	Shi470 as reference
1^b^	Jhp_0001-jhp_0021	2	HPAG1_0001-HPAG1_0023	1	HPP12_0001-HPP12_0021	1	HPSH_00005-HPSH_00120
2	jhp_0022-jhp_0042	3	HPAG1_0024-HPAG1_0045	2	HPP12_0022-HPP12_0043	2	HPSH_00130-HPSH_00240
3	jhp_0043-jhp_0298	4	HPAG1_0046-HPAG1_0317	3	HPP12_0044-HPP12_0312	3	HPSH_00245-HPSH_01635
5	Jhp_0301-Jhp_0330	5	HPAG1_0321-HPAG1_0351	4	HPP12_0315-HPP12_0350	4	HPSH_01645- HPSH_01835
6	Jhp_0333-Jhp_0413	6	HPAG1_0354-HPAG1_0436	5	HPP12_0352-HPP12_0433	5	HPSH_01850-HPSH_02260
7	Jhp_0414-jhp_0439	7	HPAG1_0437-HPAG1_0463	6	HPP12_0434-HPP12_0494	6	HPSH_02265-HPSH_02405
8	Jhp_0442-jhp_0556	8	HPAG1_0466- HPAG1_0590	7	HPP12_0498-HPP12_0618	−14	HPSH_04465-HPSH_03815
4	Jhp_0299-jhp_0300	9	HPAG1_0591-HPAG1_0594	8	HPP12_0619-HPP12_0622	−13	HPSH_03810 HPSH_03805
9	Jhp_0557-jhp_0628	10	HPAG1_0595-HPAG1_0671	9	HPP12_0623-HPP12_0700	−12	HPSH_03800- HPSH_03425
-10^a^	Jhp_0638-jhp_0631	11	HPAG1_0675-HPAG1_0682	10	HPP12_0701-HPP12_0708	−11	HPSH_03405-HPSH_03365
11	Jhp_0639-jhp_0658	12	HPAG1_0684-HPAG1_0705	11	HPP12_0709-HPP12_0729	−10	HPSH_03360-HPSH_03265
-12	Jhp_0661-jhp_0660	-13	HPAG1_0708-HPAG1_0707	12	HPP12_0731- HPP12_0732	−9	HPSH_03250 -HPSH_03245
13	Jhp_0663-jhp_0753	14	HPAG1_0710-HPAG1_0802	13	HPP12_0734- HPP12_0823	−8	HPSH_03230- HPSH_02725
14	Jhp_0757-jhp_0812	15	HPAG1_0804-HPAG1_0861	14	HPP12_0825- HPP12_0878	−7	HPSH_02720-HPSH_02420
15	Jhp_0815- jhp_0913	16	HPAG1_0863-HPAG1_0960	15	HPP12_0880- HPP12_0975	15	HPSH_04655-HPSH_05170
16	Jhp_0915	17	HPAG1_0963	16	HPP12_0979	16	HPSH_05200
17	5S-Jph_1023	18	HPAG1_r015S-HPAG1_1035	17	HPP12_5S-HPP12_1062	17	HPSH_5S-HPSH05635
18	Jhp_1024-jhp_1197	19	HPAG1_1036-HPAG1_1220	18	HPP12_1063-HPP12_1242	18	HPSH_05640- HPSH_06610
19	Jhp_1198-jhp_1206	-22	HPAG1_1235-HPAG1_1227	19	HPP12_1243-HPP12_1252	19	HPSH_06615-HPSH_06655
20	Jhp_1207	-21	HPAG1_1226	20	HPP12_1253	−20	HPSH_06665
21	Jhp_1210-jhp_1211	-20	HPAG1_1223-HPAG1_1222	21	HPP12_1256-HPP12_1257	21	HPSH_06670-HPSH_06675
22	Jhp_1212- Jhp_1261	23	HPAG1_1237- HPAG1_1289	22	HPP12_1258- HPP12_1305	22	HPSH_06685-HPSH_06935
23	jhp_1262-jhp_1282	24	HPAG1_1290-HPAG1_1311	23	HPP12_1306-HPP12_1363	23	HPSH_06950-HPSH_07060
24	jhp_1284-jhp_1285	25	HPAG1_1313-HPAG1_1316	24	HPP12_1365-HPP12_1366	24	HPSH_07085-HPSH_07095
25	jhp_1286-jhp_1295	26	HPAG1_1317-HPAG1_1327	25	HPP12_1367-HPP12_1376	25	HPSH_07100-HPSH_07150
26	Jhp_1298- jhp_1320	27	HPAG1_1330- HPAG1-1352	26	HPP12_1379 -HPP12_1399	26	HPSH_07170- HPSH_07270
-27	Jhp_1325-jhp-1322	28	HPAG1_1353-HPAG1_1356	27	HPP12_1400- HPP12_1403	27	HPSH_07275- HPSH_07290
28	Jhp_1327-jhp_1346	29	HPAG1_1360- HPAG1_1379	28	HPP12_1409 - HPP12_1430	28	HPSH_07305- HPSH_07435
29	Jhp_1347-jhp_1421	-30	HPAG1_1461- HPAG1_1383	29	HPP12_1432-HPP12_1507	29	HPSH_07445-HPSH_07875
30	Jhp_1423-jhp_1436	31	HPAG1_1464-HPAG1_1479	30	HPP12_1509-HPP12_ 1522	30	HPSH_07890- HPSH_ 07950
31	Jhp_1438-jhp_ 1441	32	HPAG1_1481-HPAG1_1484	-31	HPP12_1527 - HPP12_1524	31	HPSH_07970-HPSH_ 07985
32	Jhp_1444- jhp_1461	-33	HPAG1_1487- HPAG1_1504	32	HPP12_1529-HPP12_ 1546	32	HPSH_08005- HPSH_08100
33	Jhp_1464- jhp_1492	34	HPAG1_1505- HPAG1_ 1533	33	HPP12_1548 – HPP12_1576	33	HPSH_08110-HPSH_08250
34	Jhp_1494	1	HPAG1_1536	34	HPP12_1580-HPP12_1581	34	HPSH_08270-HPSH_08275

a. “-” indicates an inversion block.

b. All blocks on the same row are homologs.

The above result encouraged us to study genomic rearrangement, particularly inversion, of this species in more detail. There was evidence that chromosomal inversions in *H. pylori* are symmetric around the replication axis from replication origin to replication terminus [14], [44], as shown in Figure S2. The symmetric inversions around the replication origin could be explained by the process of genome replication around the replication axis when the inversions were formed [14]. Because of the difficulty to identify the inversion junctions with certainty at the boundaries of recombination in many cases in *H. pylori*, it was considered that illegitimate recombination has an important role other than homologous recombination in these genome rearrangements [14]. The homologous recombination between two inverted repeat sequences resulted in an inversion of a segment of genome in bacteria and eukaryotic cells [45], [46], [47], [48]. Currently, many *H. pylori* genome sequences are available, which facilitate the determination of inversion borders on the basis of multiple genome comparison. We found some evidence to show that homologous recombination contributed to *H. pylori* genomic inversion and illegitimate recombination was involved in the production of inverted repeats.

**Figure 2:**
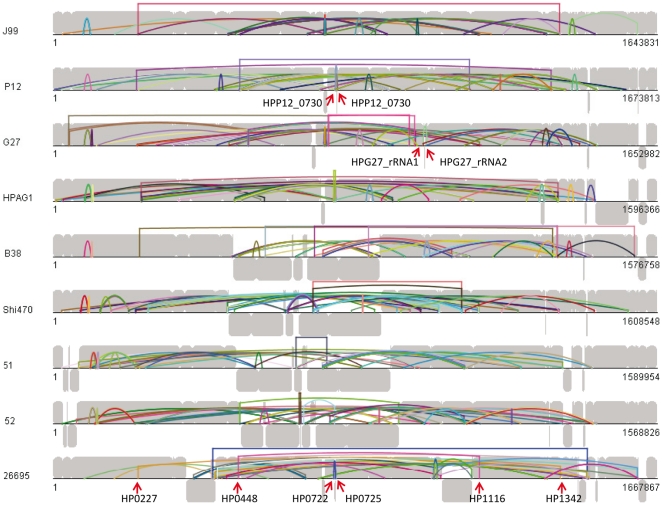
Distribution of inverted sequences on *H. pylori* genomes. The REPuter output files were combined with the Mauve alignment “coordinates output file” to make a plot with java script codes. The grey background figure shows the conserved blocks between genomes produced by Mauve. All the color lines indicate a pair of inverted repeats (>25 bp) at the position of genomes. Red arrows show the positions of some repeats mentioned in the text.

The distribution of inverted repeats (>25 bp) of nine genomes was shown in Figure 2. We first noticed the inverted rDNA repeated sequences. Each *H. pylori* strain normally has two sets of rRNA genes locating on complementary strand, of which 5S rDNAs are generally sited together with 23S rDNA to form a gene cluster. However, strains B38, G27 and 26695 have an additional 5S rDNA on sequence strand with 17 bp less in size. Further analysis showed that an inversion occurred between two 5S rDNA copies (HELPY_5S_2 and HELPY_5S_3 in B38; HPG27_rRNA1 and HPG27_rRNA2 in G27). The inverted repeat sequences are, respectively, 189 bp in B38 and 518 bp in G27, both of which exactly start from the first base of the 5S rDNAs, the junction point of the inversion. The situation in strain 26695 is more complicated. One 5S rDNA (HP_r02) has been translocated to the new site with coordinate number 448451 and 448585, comparing with genomes of G27 and B38. Some features of this new site need to be mentioned. Firstly, the rDNA cluster here is on the sequence strand, different from the region of the cluster in other genomes; Secondly, the rDNA cluster is included in a pair of inverted repeat fragments with the size of 10.5 kb (coordinate number: c1483962-1473496; 438181- 448645); Thirdly, several genes (HP0428-HP0435) at the downstream of 5S rDNA in strain 26695, has their homologs positioned (coordinate number about 1000000) within the inversion between 5S rDNA repeats in G27 and B38. The above information suggests that the novel architecture of the additional rDNA in strain 26695 should be involved in another inversion, resulting in the movement of fragments. In other words, this recurrent inversion occurred after the formation of 5S rDNA duplication in strain 26695.

**Figure 3:**
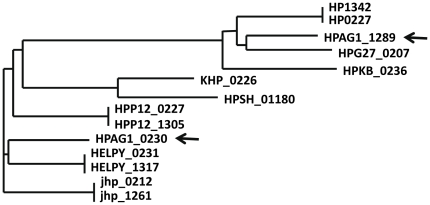
The phylogenic tree of repeats HP0227 and HP1342 with their homologs in different genomes. The arrows indicate the homologs in strain HPAG1.

The majority of inverted repeats did not lead to inversion, as shown in Figure 2. Thus, the inverted repeat sequences on genomes were further studied. The strains 26695, HPAG1, P12, J99 and B38 have two paralogs, respectively called HP0227 and HP1342, HPAG1_0230 and HPAG1_1289, HPP12_0227 and HPP12_1305, jhp0212 and jhp1261, HELPY_0231 and HELPY_1317 but the strains G27, Shi470, 51 and 52 have only one homolog. The genomic structure analysis showed that HP0227, HPAG1_0230, HPP12_0227, jhp0212, and HELPY_0231 with their neighbor genes have the similar structure to the homologs HPG27_0207 in G27, HPSH_01180 in Shi470, KHP_0226 in strain 51 and HPKB_0236 in strain 52, indicating that HP0227, HPAG1_0230, HPP12_0227, jhp0212, and HELPY_0231 are the original, and the duplications are the paralogs HP1342, HPAG1_1289, HPP12_1305, jhp1261 and HELPY_1317. The original and its paralog are the inverted repeats. Actually, the repeat sequences are a little larger than the coding region. In strain 26695, the repeat region is 2114 bp long, including the whole sequence of coding region 2076 bp. Multi-alignment showed that the original segments have two types of sequences in strains, one as representative in 26695 and the other as in P12, J99 and Shi470. The inverted sequences in each strain are almost identical, indicating that the new copy is of intragenomic origin (duplication). But the strain HPAG1contains these two types of sequences (Figure 3), implying that one of the paralogs could be of exotic origin realized by horizontal transfer. Therefore, it is very possible the first introduction of new copy to the new site that resulted in inverted repeats with identical sequences and then the occurrence of recombination to integrate exotic homologs through horizontal transfer.

**Figure 4:**
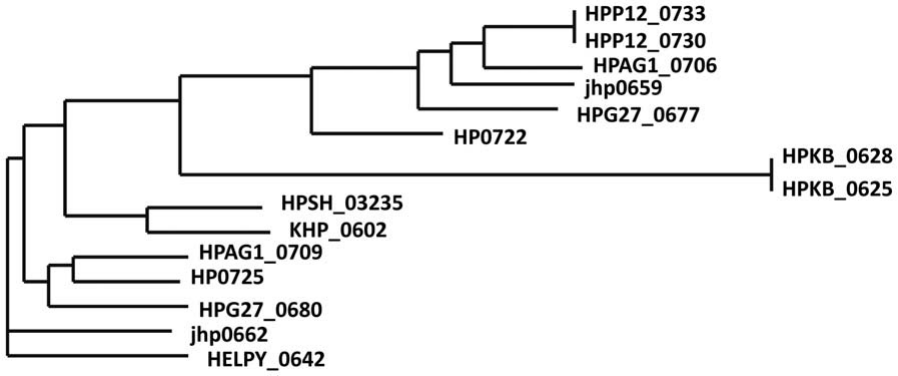
Sketch map of genomic structure in an inversion. Each arrow-formed block represents a gene open reading frame (ORF). The solid block indicates a pair of inverted repeats. The density from pale to dark shows the nucleotide identity degree of various regions between these two inverted repeats in which the dark region means identical. Small black lines indicate other genomic sequences. The letters within the blocks are the names of genes.

**Figure 5:**
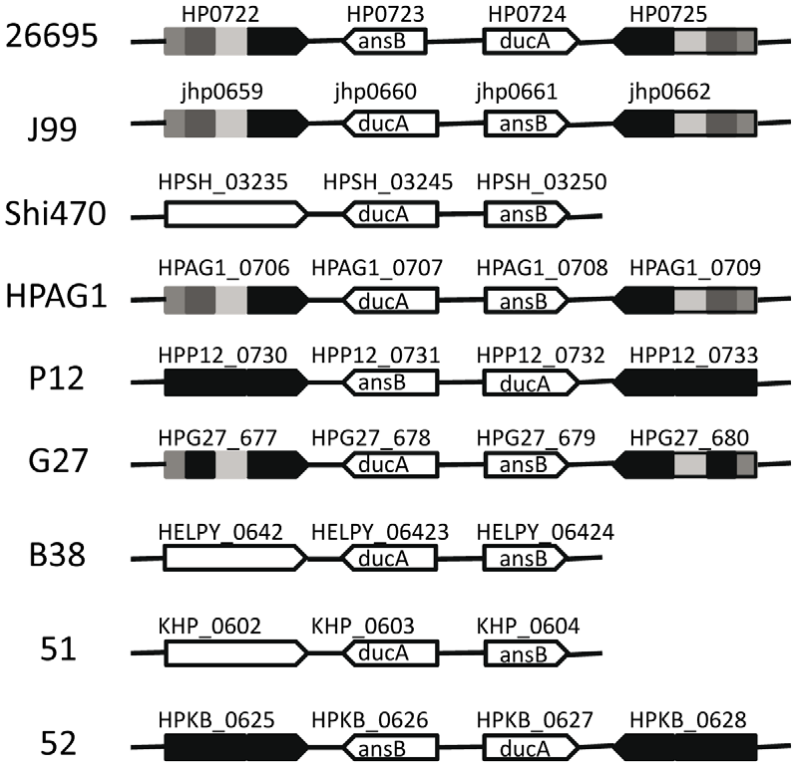
The phylogenic tree of repeats HP0722 and HP0725 with their homologs in different genomes.

The first introduction of inverted sequence can lead to the formation of a family of outer membrane proteins. In strain 26695, there are a pair of inverted repeat fragments, 2144 bp including HP0722 (omp16) on the sequence strand and 2163 bp including HP0725 (omp17) on the complementary strand. These two ORFs encode outer membrane protein with 1187 bp identical at the last half part of gene. The two genes, *ansB* (L-asparaginase II) and *dcuA* (anaerobic C4-dicarboxylate transporter) are located between them. The organization from different *H. pylori* strains are shown as Figure 4. The order of *ansB* and *dcuA* on the genomes shows, with no doubt, the presence of inversion in some strains between the inverted sequences. The strains B38 (HELPY_0642), Shi470 (HPSH_03230), and 51 (KHP_0602) have only one homolog at the similar position to HP0722 and the following genes are in the same order as *dcuA*-*ansB*. So the fragment including HP0722 is the original and the sequence containing HP0725 was the duplication while *dcuA*-*ansB* should thus be the original gene order in contrast to the inverted form as *ansB*-*dcuA*, as occurred in strains 26695, 52 and P12. In strains P12 and 52, the whole fragments of repeats are almost identical within a genome (only 1 bp deleted in one fragment in P12), which could be the consequence of recent duplication. Phylogenic analysis showed that the inverted repeat sequences at two sites within a genome have been diversified in some strains (Figure 4). The identical sequences are located at the last half part of gene but the first half part, close to the inversion junction, have been changed to great extent in various regions (Figure 5), indicating that the recombination had been involved in this region and thus a family of proteins were created. The diversification of paralogs after duplication could be similar to the case of *homA* and *homB* shown in the recent report [49]. On the other hand, the borders of inversion are the inverted repeats, as the case of 5S rDNA inversion mentioned above, indicating that these inversions could be realized by homologous recombination through inverted repeats. We further compared the junction sequences of the inverted repeats and found that the exterior border sequences (16–21 bp) of the junctions could be the same from different strains but never in the same strain (more than 70% of identity) even though the repeats within a genome are identical, suggesting that the inverted repeat could be introduced by illegitimate recombination. To this sense, the process of inversion could be the first introduction of the inverted repeat to a new site by illegitimate recombination and then the occurrence of inversion between two inverted repeats. It can also explain why the strains with inverted repeats (strains J99, G27, and HPAG1) still kept the same gene order between the inverted regions as the strains without the inverted repeats (strains 51, Shi470 and B38): only occurred the inverted duplication and the inversion did not happen yet. Of course, an extreme case could occur that the inversions happen in even number of times.

Another case showed that the inverted duplication resulted in the generation of mosaic or new genes. HP0448 and HP1116 were annotated as mosaic genes due to the existence of shared identical sequence of 1430 bp. Genomic comparison between strains showed that: other strains have the same genomic structure flanking the orthologs of HP0448; HP1114, HP1117 and their orthologs are conserved in different strains but the sequences between them are distinct, indicating no consistent genomic structure around HP1116; the 1430 bp-repeated fragment within HP0448 or its orthologs in other strains are usually sited at the opposite orientation to those paralogs, such as the repeats within HPKB0849 or HP1116, representing that they are inverted repeats within a genome. In B38, two identical fragments (1426 bp) within pseudogenes HELPY_0867, HELPY_0866 and HELPY_1087, HELPY_1086 are in the same direction. However, there is still a residue segment (468656–468800) that corresponds to the first 145 bp of the 1430 bp-repeated fragment and locates at similar place in the same orientation as HP0488, indicating that the 1430 bp-repeated segment ever existed here. We thus concluded that the fragment in HP0448 is the original and the others are inverted copies. Because of the repeat fragments within the gene, the duplication in new sites caused the creation of new ORFs or mosaic genes.

As shown in Figure 2, many inverted repeats did not cause inversions. However, the inverted repeat sequences provided the sources of inversions. In B38, five inverted largest sequences are transposon 609 (2.4 kb), four of which are identical except 1 bp difference in one transposon (1318401–1316023), indicating the transpositions occurred recently and they are still active [31]. Because of one transposon located at the opposite direction, four pairs of inverted repetitions are thus formed. No inversions have been found in the strain involved in the four pairs of repeats but it could happen in the population. Meanwhile, some inversion could be limited if the inversion occurs within a gene (ORF, promoter), within an operon, or the inversion changed the location of origin or terminus of replication. Actually, the border sequences of inversion are complicated and difficult to analyze in *Helicobacter* genomes if the inversion is produced by illegitimate recombination or by small repeats-mediated homologous recombination because of the problems on the low similarity and the resolution of multi-alignment. However, it is a certainty that the homologous recombination has an important contribution in the formation of genomic inversion and illegitimate recombination participated in the occurrence of both genomic inversion and inverted repeats.

### Core genome and the core genes of particular adaptation to human host

**Figure 6:**
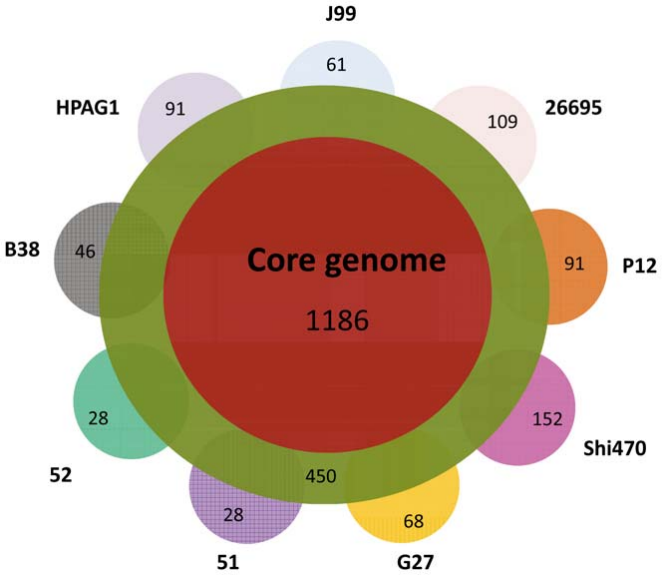
Sketch map of core genome for protein-coding genes. The central cycle represents core genome. The secondary cycle indicates the genes that are shared by at least two genomes. Other small partial cycles show the strain-specific genes. The number within the cycles means the number of genes in this category.

A set of genes that are present in all the strains of a species is referred as a core genome to the species. The genomes that are currently available in databases were sampled from Asia, Southern and Northern America, Europe. The strain J99 was identified as Africa-type strain. So the new core genome produced on the basis of current genomic information should be more representative than ever before. Here we used the program OrthoMCL to compare the protein sequences of each protein-coding gene (see Materials and Methods). The 1171 genes were found to exist in all nine strains. And then this set of genes were compared with the core genome, consisting of 1150 protein-coding genes, that was produced on the microarray data of 56 clinic strains [50]. There were 197 proteins not in common between them, of which 107 proteins were identified from our analysis and 90 proteins from the published data. To understand the reason of this difference, each of incongruent genes were manually evaluated. The sequences of both DNA and proteins were used to evaluate all the incongruent genes in our comparison. Thus, we first tested protein sequences of orthologs from different strains. If it was difficult to make a decision, the DNA sequence was further tested. In this case, we used one gene sequence to do the Blast searching for getting the corresponding sequences in other strains for multi-alignment. The results showed that: 122/197 genes have been accepted as part of core genome; we lost four genes that have not been annotated in some strains, including two ribosomal proteins, L34 (HP1447) and L36 (HP1297); most of false core genes came from the published data, including pseudogens particularly due to frameshifts in some strains and size-different genes. As for the gene HP0326, it is a two-gene fusion. In B38, these two genes were called as HELPY0329 and 0330. Because the genome sequence of strain 26695 was sequenced first in this species, all the following annotation considered this sequence as a frameshift mutation. Actually, HP0326 has a nucleotide “C” insertion at the position 626 of DNA sequence to lead to gene fusion. All other strains tested here contained two genes that have 7 nt overlapped. That is why the fusion gene and two overlapped genes have a good alignment even with high identity. We checked the orthologs of all sequenced bacterial genomes hitherto in GenBank and found that only a few of the orthologs were fused. Thus, these two genes were considered as members of core genome. The core genome as 1186 protein-coding genes was finally obtained, as shown in Figure 6 and Table S2.

Core genes are the consequence of adaptation to specific niche of species. Human is the natural host for *H. pylori*. The composition of *H. pylori* core genome should be the consequence of co-evolution between pathogen and its human host. Sequence analysis showed that *H. acinonychis* is a bacterium that shares the same ancestor with *H. pylori* and was transferred from early human to carnivorous animals [51]. It means that these two species were diversified from the same origin and were limited by different niches (human or animal stomachs). Therefore, some core genes in *H. pylori* could be not present or mutated in *H. acinonychis* after host shift, furthermore, implying that these core genes should be important for *H. pylori* life in human host and may be the candidate targets for chemoteraphy. We then compared this core genome with the genome of *H. acinonychis* sheeba and found that 22 core proteins were only occurred in *H. pylori* genome but they were not present or strongly fragmented in *H. acinonychis* (Table 2). These proteins came from four categories, outer membrane proteins, metabolic proteins, DNA modification proteins and some uncharacterized proteins.

**Table 2:** *H. pylori* core genes that were not present or not functional in *H. acinonychis* Sheeba genome.

Proteins	Functions
**hypothetical protein**	
HP0614	Hypothetical protein
HP0902	Hypothetical protein
HP1580	Hypothetical protein
HP1588	Hypothetical protein
**membrane protein**	
HP0189	UPF0114 protein, Transmembrane
HP0209	Putative uncharacterized protein
HP0227	Outer membrane protein (Hop, Omp5)
HP0228	Conserved hypothetical integral membrane protein
HP0229	Outer membrane protein (Omp6)
HP1177	Outer membrane protein (Omp27)
HP1502	Conserved hypothetical integral membrane protein
**metabolic proteins**	
HP0208	Lipopolysaccharide 1,2-glycosyltransferase
HP0211	HcpA
HP0357	Short chain alcohol dehydrogenase
HP0407	Biotin sulfoxide reductase (BisC)
HP0473	Molybdenum ABC transporter, periplasmic molybdate-binding protein (ModA)
HP0475	Molybdenum ABC transporter, ATP-binding protein (ModC)
HP0798	Co-factor de molibdeno en la bio-síntesis de proteína C (MoaC)
HP0800	Molybdopterin converting factor, subunit 2 (MoaE)
HP0814	Thiamin biosynthesis protein (ThiF)
**DNA modification**	
HP0478	Adenine specific DNA methyltransferase (VspIM)
HP0854	Guanosine 5′-monophosphate oxidoreductase

HP0211 (HcpA), a cysteine-rich protein A, called as beta-lactamase [52], is a conserved protein with the same size, 255 amino acids (aa), in all *H. pylori* strains tested. It is a secretion protein containing the signal peptide and 5 tratricopeptide repeats (TPR repeat seems to be responsible for the protein-protein interaction). It was confirmed that HcpA protein in *H. pylori* is expressed under natural environmental conditions and is recognized by the immune system of human due to HcpA antibody present in sera from human patients infected by *H. pylori* [53]. This protein slowly hydrolyzes 6-aminopenicillinic acid and 7-aminocephalosporanic acid (ACA) derivatives. So it may be involved in the synthesis of the cell wall peptidoglycan to participate in the antibiotic resistance process [52]. Each *H. acinonychis* genome normally has several cysteine-rich proteins. A recent report further showed that it is HcpA (HP0211) but not HcpC (HP1098) to cause the differentiation of human myeloid Thp1 monocytes into macrophages. Thus, HcpA is a bacterial immune modulator on the process of infection [54].

HP0357 is a short chain alcohol dehydrogenase. *H. acinonychis* has not the homologous sequence but in *H. pylori* it is a conserved protein with 250–253 aa. This protein has two domains, one for binding the coenzyme and the other for binding the substrate. This latter domain determines the substrate specificity and contains amino acids involved in catalysis as the active center. HP0357 contains the typical sequence as YxxxK at the amino acid position 149–152 (active center) and the sequence as SGxxxGxG at the position 8–14, similar to the typical sequence TGxxxGxG of N-terminal cofactor binding site [55]. Further analysis showed that 18th amino acid downstream of the third glycine residue (G) is not aspartate residue (D) but another G, indicating that this protein possesses a preference for NADP(H) over NAD(H) [56]. The short-chain alcohol dehydrogenases participate to synthesize a variety of intercellular signals and other chemically diverse products. In *Myxococcus xanthus*, the *csgA* gene encoding for the short-chain alcohol dehydrogenase protein was responsible for the manifestations of C signaling. The C signal is a concentration-dependent developmental timer that controls spatial and temporal gene expression with the emergence of the morphologically distinct development [57], [58]. More researches on the functions of this protein in *H. pylori* are needed.

The molybdenum is essential for the majority of microorganisms. Several core genes (Table 2) are involved in the bacterial molybdenum uptake and the molybdenum cofactor biosynthesis. A specific ATP-binding cassette (ABC) transporter consisting of proteins ModABC is responsible for the transfer of molybdate (MoO_4_^2−^), the bioavailable type of molybdenum in nature. ModA is a periplasmic molybdate-binding protein that captures molybdate and then transfers to ModB, the transmembrane channel with the help of ModC, an ATP-binding protein. In *H. pylori*, the gene together with ModA and ModB in the operon was annotated as ModD. We suggest this gene should be annotated as ModC according to the genomic structure and their functions. A complete series of proteins, MoaA, C, D, E; MoeA, B; MogA and MobA, related to molybdenum cofactor biosynthesis exist in most of *H. pylori* strains. In the strains 51 and 52, they lost some genes such as ModB and MoeA. *H. acinonychis* did not have all the genes mentioned above. How they utilize molybdenum for their molybdenum-dependent enzymes is still yet unknown. They may adopt another pathway to make use of molybdenum.

HP0814 is an enzyme (ThiF) that catalyzes the adenylation of ThiS, as part of the biosynthesis pathway of thiamin pyrophosphate (vitamin B1). ThiF belongs to the family of E1-like enzymes and ThiS is its substrate. Therefore, *thiF* and *thiS* generally occur as an operon. However, all the strains detected in *H. pylori* have the conserved *thiF* without the homolog of *thiS* on their genome. To this sense, ThiF may have different function in *H. pylori*.

HP0478 and HP0854 are involved in DNA modification. HP0478 belongs to type II adenine specific methyltransferase (VspIM), which functions as N-6 DNA methyltransferase and recognizes the sequence ATTAAT [59]. HP479 could be its R gene due to the existence of gene fusion of homologous HP0478 and HP0479 sequence in strain Shi470. HP0854 is a guanosine 5′-monophosphate oxidoreductase (GuaC), which catalyzes the irreversible NADPH-dependent deamination of GMP to IMP, functioning in the conversion of nucleobase, nucleoside and nucleotide derivatives of G to A nucleotides, and in the regulation of intracellular balance of A and G nucleotide. *H. pylori* core genome also contains the gene *guaA* (HP0409) and guaB (HP0829), *purA* (HP0255) and *purB* (HP1112), all of which are related to purine metabolism. However, the yeast two-hybrid tests showed that HP0854 interacts with another core protein HP0377, thiol-disulfide interchange protein (DsbC) [60]. HP0377 contains the conserved active motif FxxxxCxxC as that in *E. coli*, implying that its function in vivo could be in the formation of disulfide bonds in proteins [61]. This new interaction between HP0854 and HP0377 could indicate new function.

Some membrane proteins have been fragmented or do not exist in *H. acinonychis*. HP1502 is an integral membrane protein, predicted belonging to the protein of unknown function DUF474 family (InterPro). HP1502 and its homologs have 145aa in all strains tested. HP0189 is uncharacterized transmembrane proteins, belonging to the UPF0114 family. It is a conserved protein as the sizes 177–178 aa in *H. pylori*. *H. acinonychis* only has the last half part (89 aa) (Hac_0374), keeping the last transmembrane motif. Protein-protein interaction experiments showed that it interacts with urease accessory protein UreI [60], participating in the regulation of environmental pH. *H. acinonychis* did not have the homologous DNA sequence of HP0209, which is still an uncharacterized protein in *H. pylori*. HP0227 is an outer membrane protein with sizes 691–721 aa, except for the 633 aa homolog in B128 and 658 aa of a copy in HPAG1, lost the last part. As mentioned before, several strains have the second copy. HP0227 showed the interactions with HP1259, HP1382 and HP1427 [60]. HP1427 is a histidine-rich, metal binding polypeptide (Hpn), interacting with both membrane proteins HP0227 and HP0229 [60]. HP0228 is an integral membrane protein, belonging to xan_ur_permease superfamily for the transport of diverse substrates such as xanthine, uracil, and vitamin C. HP0228 also contains sulfate permease motif. It is actually uncharacterized functionally for its substrate in *H. pylori*. However, it showed the interaction with HP0016 [60]. It is also possible to function together with carbonic anhydrase (IcfA) (HP0004) because these two homologous genes were fused in *Mycobacterium tuberculosis*. HP0227, HP0228 and HP0229 occurred collectively and shared some protein interaction, indicating that they could work coordinately for a specific function. Another protein is HP1177 (*hopQ*), which homologous sequence has been fragmented into four parts in *H. acinonychis*. Two-hybrid test showed that it interacts with HP0241, another hypothetic protein [60].

Four hypothetical proteins are not present or partially present in *H. acinonychis* but exist in all the *H. pylori* strains tested. HP0614 and its homologs in *H. pylori* strains are in the same size of 110aa while the homolog in *H. acinonychis*, Hac_1390, only has its partial sequence, 74aa. No clear motif has been detected. HP0902, present in the size of 99aa in all *H. pylori* strains, contains cupin domain, belonging to Cupin_2 family. This family represents the conserved barrel domain consisting of beta bands. The interactions with three proteins HP0887 (vacuolating cytotoxin precursor), HP0588 (ferrodoxin-like protein), HP1409 (hypothetical protein) was demonstrated by two-hybrid test [60]. HP1580, an uncharacterized protein and its *H. pylori* homologs in other *H. pylori* strains have been annotated in two sizes in 198 aa and 220aa (strains 26695, P12, 51 and HPAG1). Actually, they have the same size if they are annotated from the same start codon (both are ATG). HP1580 is type 2 phosphatidic acid phosphatase (PAP2) _like_5 domain, which may act as a membrane-associated lipid phosphatase. HP1588 and its homologs are much conserved with the same size as 253aa in *H. pylori*. It contains the motif of ubiquinol-cytochrome C chaperone, which is required for assembly of coenzyme QF-2-cytochrome C reductase in yeast [62].

*H. acinonychis* is a closely related species to *H. pylori* but lives in feline animal stomach. Comparing the core genome of *H. pylori* with *H. acinonychis* genome is logical to find genes that particularly adapts to human stomach. The 22 genes shown here, except four uncharacterized genes, are involved in bacterial activity to challenge the environment, indicating that our analysis is effective. We know that only one genome from *H. acinonychis*, which can be available so far, as a reference is not sufficient to make definite decision for adaptation but it provides a possible way to screen the adapted genes to human host. Because of the analyzed *H. pylori* strains isolated from different continents, the 22 genes, even though they may not be present in all the clinic strains, should extensively exist in *H. pylori* strains and could have important contribution to adaptation. In addition to the functional confirmation of HP0208 [63], nine of the genes have also been demonstrated to be involved in the progress to chronic atrophic gastritis (orthologs: HPAG1_0785-HP0800; HPAG1_0783-HP0798; HPAG1_1035-HP0357; HPAG1_0985-HP0407; HPAG1_1536-HP1588; HPAG1_0212-HP0211; HPAG1_0452-HP0475; HPAG1_0455-HP0478; HPAG1_0838-HP0854) [28]. Most of these gene functions are not yet known in *H. pylori* and need to be clarified in the future research.

### Pseudogenes and homopolynucleotide

Pseudogenes consist of high proportion in *Helicobacter* bacteria. *H. acinonychis* has the highest percentage and *H. pylori* genomes have 5.6–8.7%. *Helicobacter* genomes that kept so high pseudogenes should have their adaptive significance. We noted that dominant proportion of pseudogenes in *H. pylori* strains kept the whole orthologous sequences of genes, implying that they were recent mutations. Some evolutionary implications could be thus derived from the structural features of pseudogenes by sequence comparison. In *H. acinonychis* genome, 92 genes present in *H. pylori* were fragmented into 255 orthologous coding sequences, showing the new adaptation as the latest ancestor changed the host from human to feline animals [51]. However, it is difficult to infer more information on recent *H. pylori* evolution from the *H. acinonychis* mutations because the sequences have been changed to a great extent.

**Figure 7:**
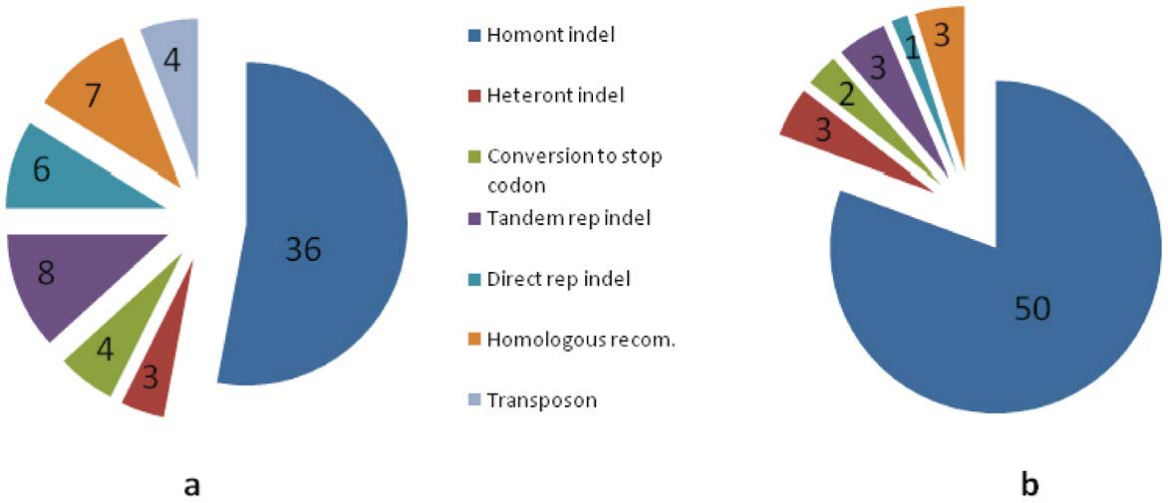
The portions of types of premature mutations in pseudogenes. a. premature mutations in pseudogenes in strain B38. b. premature mutations in pseudogenes in other strains.

Therefore, that *H. pylori* genomes possess the high proportion of pseudogenes in the intraspecies strains is an ideal model for detecting the recent genomic evolution of species. We firstly analyzed the pseudogenes in B38. The 59 fragmented genes (28 hypothetic proteins, 11 membrane proteins, 11 restriction-modification system proteins and 9 others) were chosen for comparison because the orthologous sequences exist in other strains. The multi-alignment analysis of the 59 pseudogenes demonstrated that the mutations were generally resulted from frameshift: except 4 genes interrupted by ISHp609 and 4 gene with the occurrence of new stop codon by point mutations, 60 premature mutations were formed by frameshifts (Figure 7a, Table S3). These frameshifts were caused by indels of homonucleotides, heteronucleotides, short tandem repeats, short direct repeats and recombination with other fragments. However, the indel of homonucleotides were the principal contribution, 60% of total frameshifts (36/60). As the consequence of such indels, one or more nucleotide differences existed at the mutation sites of homopolynucleotide (HPN) when compared to normal sequences. We further analyzed the 50 pseudogenes that were randomly selected from other strains and showed that up to 90% of prematures were responsible for homonucleotide indel (Figure 7b. Table S4).

**Figure 8:**
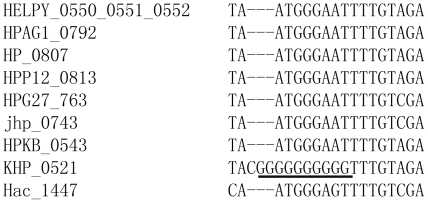
Creation of homopolynucleotide (HPN) possibly by recombination. The left is the locus-tag of gene. The right is the partial sequences of gene.

It is reasonable to explain it as the result of single-strand slipped repair and lack of some genes for DNA repair and recombination system in *H. pylori* [11], [25], so that the short HPN could produce longer one by this mechanism and *vice versa*. Similarly, frameshift mutations caused by the short tandem repeats from 2 bp to 106 bp also existed in pseudogenes (Table S3). On the other hand, recombination could also participate in this process. The multi-alignment of pseudogenes HELPY0550, 0551, 0552 with other orthologous sequences gave a good example, as shown in Figure 8. The formation of 10 guanine HPN could not be explained by point mutation or from the 3-guanine nucleotide sequence by single-strand slipped repair but was most possibly created by a step of recombination with other fragment by the mechanism of illegitimate recombination if the mutation was *de novo* in the population. Although this insertion caused an inframe mutation here, the principle for introduction of long homonucleotide sequence leading to frameshift could be the same mechanism, implying that the HPN can be formed at a specific site in one-step by both single-strand slipped repair or by recombination.

The genes with long HPN sequences at mutation sites, generally more than or equal to 7 nucleotides, are possible contingency genes [22]. In our study, some mutations at the sites with more than or equal to 7 HPNs only occurred in B38. For example, the pseudogene including sequences of HEPLY0550, 0551 and 0552 had 8(A) (a HPN composed of eight adenines) at the mutation site but three types of normal sequences existed repectively in other strains (7(A), GCAAAAA and ACAAAAA). Meanwhile, all the frameshift mutations caused by less than 7 bp HPN in B38 kept the normal sequences at the place in other strains or the similar mutations present in other sites of gene (HPN mutations were not at the same place in other strians). In other words, the most framshift mutations present in our study were generally not phase-variation expression genes. In this sense, it suggests that frameshift mutations resulted from slippage repaire mediated by HPN were common events, and the contingency genes were a special case in the formation of HPN under selection pressure because they just have longer such sequence with more possiblity of unstability although this unstability could be different under specific micro-environmental condition of cells and specific strains.

The present analysis showed that HPN-mediated frameshift mutations occurred more common in the genes that contained high content of HPN, principally including the genes involved in restriction and modification system, membrane-associated proteins, lipopolysaccharides and some hypothetic proteins (Table S3 and S4). It means that the number of HPN of a gene could have relation to the HPN-mediated framshift mutations. Actually, some genes with high HPN in *H. pylori* have been mentioned by Saunders *et al* as early as 1998 [64]. At present, as much more genome sequences are available, we can quantify it in more detail.

**Figure 9:**
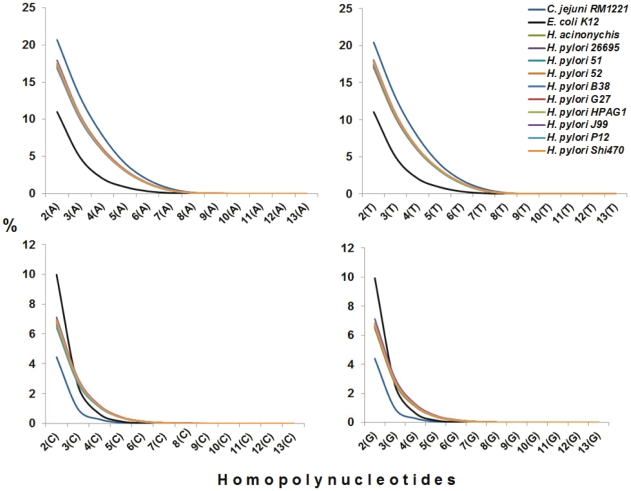
The comparison of homopolynucleotide (HPN) content in *H. pylori* strains with other closely related species.

**Figure 10:**
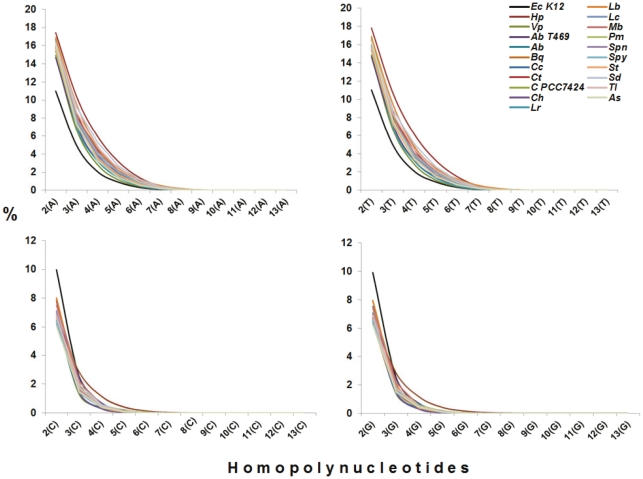
The comparison of homopolynucleotide (HPN) content in *H. pylori* 26695 with other bacteria, from Archaea to Eubacteria species. The abbreviations are: *Ab*T469, *Aciduliprofundum boonei* T469; *Ab*, *Acinetobacter baumannii* AB0057; *Bq*, *Bartonella quintana*; *Cc*, *Chlamydophila caviae* GPIC; *Ct*, *Clostridium therm*ocellum ATCC 27405; *C*PCC 7424, *Cyanothece sp.* PCC 7424; *Ch*, *Cytophaga hutchinsonii* ATCC 33406; *Lr*, *Lactobacillus reuteri* DSM 20016; *Lb*, *Leptospira biflexa* serovar Patoc; *Lc*, *Leuconostoc citreum* KM20; *Mb*, *Methanosarcina barkeri*; *Pm*, *Proteus mirabilis strain* HI4320; *Spn, Streptococcus pneumoniae* ATCC; *Spy*, *Streptococcus pyogenes* M1 GAS; *St*, *Streptococcus thermophilus* CNRZ1066; *Sd*, *Sulfurospirillum deleyianum* DSM 6946; *Tl*, *Thermotoga lettingae* TMO; *Vp*, *Veillonella parvula* DSM 2008; *As*, *Aliivibrio salmonicida* LFI1238; *Cj*, *Campylobacter jejuni* RM1221; *Ec*K12, *Escherichia coli* K12; *Hp*, *Helicobacter pylori* 26695.

High number of HPNs in genes should elevate the high HPN proportion in a genome. If this genomic feature is true in *H. pylori*, it can be extrapolated that all the *H. pylori* strains should share this feature and the genomes of *H. pylori* have at least much higher proportion of HPN than other bacterial genomes that contain the similar G+C content. Following this hypothesis, we performed the analysis in two steps: firstly, to compare all *Helicobacter pylori* genomes with the genomes of closely related species and *E. coli;* and then to compare a *Helicobacter pylori* genome with other bacterial genomes from Archaea to Eubacteria species that possess the 38.5–39.5% of G+C content. The G+C contents of 51 genomes were located within this range in the NCBI database. One representative of each species (20 species) was chosen for comparison so that 20 species genomes as well as *E. coli* were tested. The result demonstrated that all *H. pylori* strains had a similar proportion of HPN number (Figure 9). When compared to closely related species, the genomes of *H. pylori* as well as *H. acinonychis* had higher HPN proportion in all four bases, starting from tri-homopolynucleotides, indicating that it could be the common trait of *Helicobacter* genus. *Campylobacter jejuni* genome, with 30.5% of G+C, had much higher poly-A and poly-T than *H. pylori* genomes but had much less poly-G and poly-C so that its HPN composition was strongly influenced by the lower G+C content of genome. In comparison with *E. coli,* it clearly showed this feature, higher HPN in all four types of bases in *H. pylori*, including poly-G and poly-C even though *E. coli* has much higher G+C content (50.8%). When comparing with other species that contain similar genomic G+C content, it also showed the similar result, much higher HPN from tri-homopolynucleotides in *H. pylori* (Figure 10).

Therefore, the pseudogenes in *H. pylori* generally possess the characteristics: 1. high number of HPN; 2. maintenance of completely coding sequences in most pseudogenes; 3. the dominant shift mutations caused by HPN. As mentioned above, length of HPN can be changed by single-strand slipped repair so that such inframe or frameshift mutations are reversible. The pseudogenes still kept the coding sequence, indicating that it is not only the recent occurrence of events but also the possibility of reverse mutation for on-off switch expression of a gene or a partial structure of protein that could lose some functional domain if the framshifts just occurred before the domain of genes. Contingency genes seem to have hot point of mutation that could be caused by their instability due to longer HPN and functional limitation under a particular niche. In contrast to contingency genes, other HPN frameshift mutations, generally for less length of HPNs, have not been fixed to a specific site of DNA and may be slowly to restore its function, but they could be the complementary materials in the reservoir of adaptation in addition to rapid on-off switch to environmental changes such as phase variation. Slippage repair provided the chance to create HPN and the absence of some enzymes of this system in *H. pylori* could promote this process. However, the most important factor for keeping high number of HPN of a genome is that the HPN is a favorable adaptation to this species so that it could accumulate HPN sequences for its genome until the formation of equilibrium between functional limitation and the indel number of HPN.

### Conclusion

We attempted to understand the genomic microevolution of *H. pylori* from different angles for providing new insight into its special capability for adaptation. Inversion has an important role in genomic dynamics of *H. pylori*. *H. pylori* inversions are usually symmetric around the replication axis, which are realized not only by illegitimate recombination and but also by homologous recombination when two inverted repeat sequences exist on the chromosome. Inversion is a historical event, which can be used to trace the ancient genome structure. On the other hand, the formation of inverted repeats seems to be related to illegitimate recombination. The event of inversions and the occurrence of inverted repeats both contributed to the creation of new genes or gene families. The core genes that were not present or deadly fragmented in *H. acinonychis*, one closely related species that was evolved from *H. pylori* due to host shift from human to feline animal, should particularly adapt to human stomach, although many functions of these genes need to be clarified in the future. *H. pylori* pseudogenes that possess reversible mutations and keep the completely coding sequence would be a reservoir of functional genes for challenging new niche. HPN is an important factor for on-off switch of pseudogenes, particularly of contingency genes. High number of HPN in *H. pylori* genome conforms to this mechanism.

## Supporting Information

Figure S1 **Inversions shown by genome structure comparison.** The figure was produced from program Mauve. The black lines and arrows indicate the fragment rearrangements of *H. pylori* genomes.
(TIF)

Figure S2 **Symmetric structure around the replication axis on chromosome.** This figure was produced from program MUMmer. The eight *H. pylori* genomes were compared with the genome of J99. The solid line indicates the position of replication origin and the dash line shows the possible replication terminus.
(TIF)

Table S1 **The core genes used for phylogenic analysis.**
(DOCX)

Table S2 **The composition of core genome of** ***Helicobacter pylori*** **.**
(DOCX)

Table S3 **The mutations responsible for protein premature in strain B38.**
(DOCX)

Table S4 **The mutations responsible for protein premature in other genomes.**
(DOCX)
