# Correction: New Implications on Genomic Adaptation Derived from the *Helicobacter pylori* Genome Comparison

**DOI:** 10.1371/annotation/d8fae120-5f86-4df7-8e53-46afa9c01be0

**Published:** 2011-03-04

**Authors:** Edgar Eduardo Lara-Ramírez, Aldo Segura-Cabrera, Xianwu Guo, Gongxin Yu, Carlos Armando García-Pérez, Mario A. Rodríguez-Pérez

The legends for Figure 4 and Figure 5 are reversed.

