# Correction: New Implications on Genomic Adaptation Derived from the *Helicobacter pylori* Genome Comparison

**DOI:** 10.1371/annotation/2f2953da-1918-4068-a730-46660d43e6b5

**Published:** 2011-03-17

**Authors:** Edgar Eduardo Lara-Ramírez, Aldo Segura-Cabrera, Xianwu Guo, Gongxin Yu, Carlos Armando García-Pérez, Mario A. Rodríguez-Pérez

The wrong figure is cited in paragraph six, sentence five of the "Inversions and Inverted sequences" section of Results and Discussion. The sentence "The organization from different H. pylori strains are shown as Figure 4." should read "The organization from different H. pylori strains are shown as Figure 5."

