# Supplementary figures and images for: New Implications on Genomic Adaptation Derived from the Helicobacter pylori Genome Comparison
Source: PLoS One. 2011 Feb 28;6(2):e17300. doi: 10.1371/journal.pone.0017300 (PMC3046158; doi:10.1371/journal.pone.0017300)

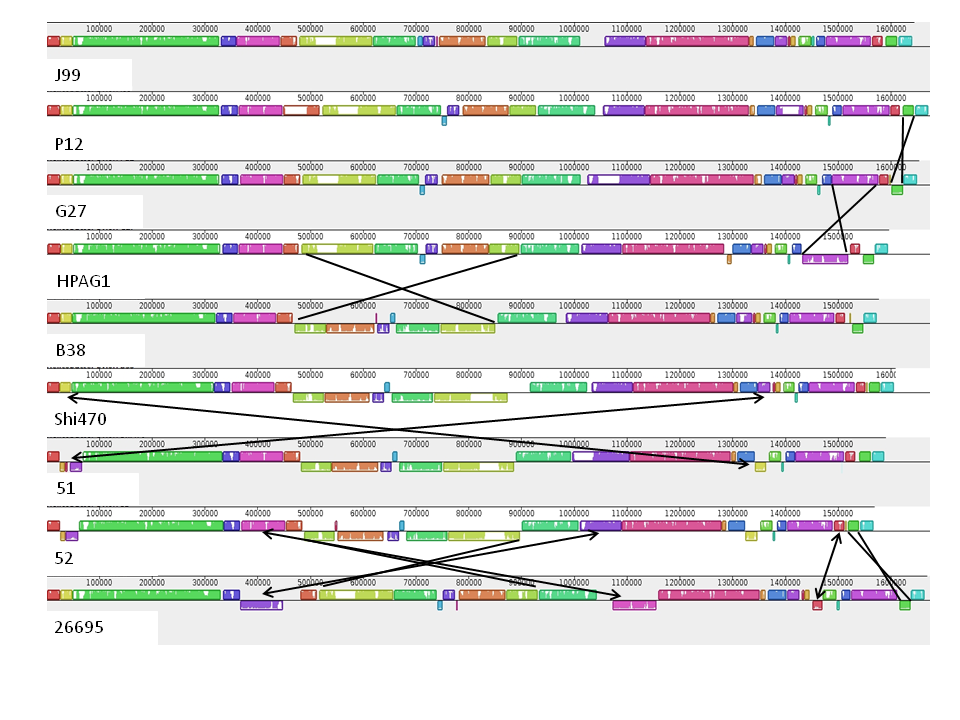

Supplement: Figure S1 — Inversions shown by genome structure comparison. The figure was produced from program Mauve. The black lines and arrows indicate the fragment rearrangements of H. pylori genomes. (TIF) [file pone.0017300.s001.tif]

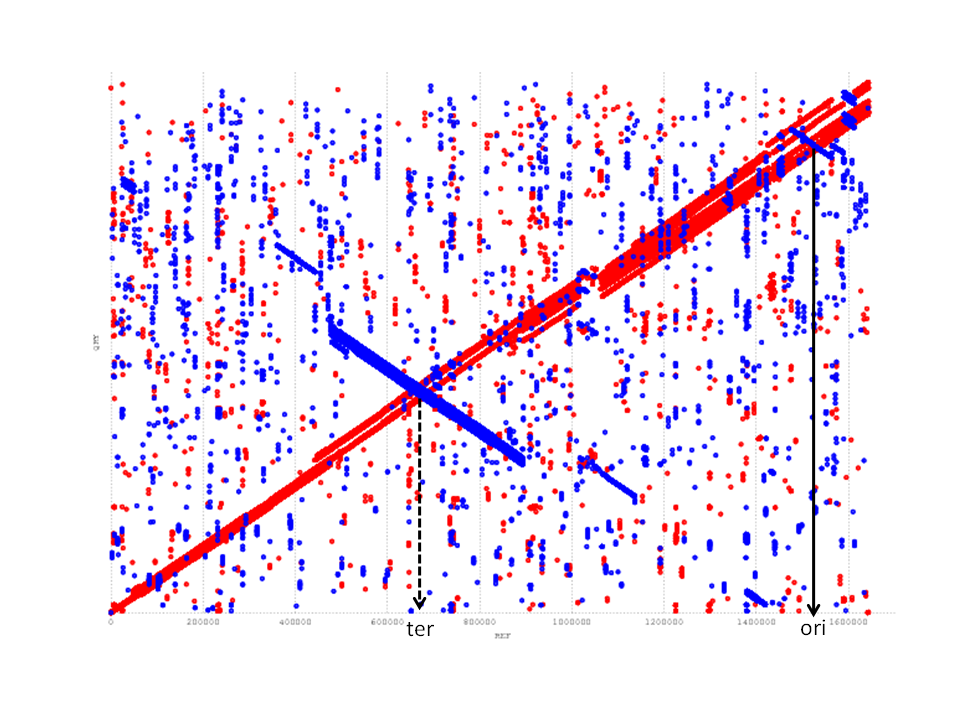

Supplement: Figure S2 — Symmetric structure around the replication axis on chromosome. This figure was produced from program MUMmer. The eight H. pylori genomes were compared with the genome of J99. The solid line indicates the position of replication origin and the dash line shows the possible replication terminus. (TIF) [file pone.0017300.s002.tif]
