# Supplementary material for: New Implications on Genomic Adaptation Derived from the Helicobacter pylori Genome Comparison
Source: PLoS One. 2011 Feb 28;6(2):e17300. doi: 10.1371/journal.pone.0017300 (PMC3046158; doi:10.1371/journal.pone.0017300)
Supplement: Table S1 — The core genes used for phylogenic analysis. (DOCX) [file pone.0017300.s003.docx]

**Table S1.** The core genes used for phylogenic analysis

| Groups | Genes |
| --- | --- |
| 1 | *minC, minD, minE, ftsZ, ftsA, ftsW, mreB, efp, aroE, folD* |
| 2 | *dnaA, dnaJ, dnaK, glnA, groEL, atpA, mutY, trpC, ureI, cysS* |
| 3 | *fic, maf, mpr, ppa, spoOJ, ureE, ureG,ureH, ylxH, lysS* |
| 4 | *engB, fbcF,fbcH, fbcHB,flgB,flgC,fliE, folC, ftsl,tagE* |
