# Supplementary material for: New Implications on Genomic Adaptation Derived from the Helicobacter pylori Genome Comparison
Source: PLoS One. 2011 Feb 28;6(2):e17300. doi: 10.1371/journal.pone.0017300 (PMC3046158; doi:10.1371/journal.pone.0017300)
Supplement: Table S2 — The composition of core genome of Helicobacter pylori . (DOCX) [file pone.0017300.s004.docx]

**Table S2.** The composition of core genome of *Helicobacter pylori*

| **Locus-tag number** |
| --- |
| HP0019 |
| HP0020 |
| HP0021 |
| HP0022 |
| HP0025 |
| HP0026 |
| HP0027 |
| HP0028 |
| HP0029 |
| HP0031 |
| HP0032 |
| HP0033 |
| HP0034 |
| HP0035 |
| HP0036 |
| HP0037 |
| HP0038 |
| HP0039m |
| HP0042 |
| HP0043 |
| HP0044 |
| HP0045 |
| HP0047 |
| HP0048 |
| HP0049 |
| HP0050 |
| HP0055 |
| HP0056 |
| HP0057 |
| HP0067 |
| HP0068 |
| HP0070 |
| HP0071 |
| HP0072 |
| HP0073 |
| HP0074 |
| HP0075 |
| HP0076 |
| HP0077 |
| HP0080 |
| HP0082 |
| HP0083 |
| HP0084 |
| HP0085 |
| HP0086 |
| HP0087 |
| HP0088 |
| HP0089 |
| HP0090 |
| HP0092 |
| HP0095 |
| HP0096 |
| HP0097 |
| HP0098 |
| HP0099 |
| HP0100 |
| HP0101 |
| HP0102 |
| HP0103 |
| HP0104 |
| HP0105 |
| HP0106 |
| HP0107 |
| HP0109 |
| HP0110 |
| HP0111 |
| HP0112 |
| HP0113 |
| HP0114 |
| HP0115 |
| HP0116 |
| HP0117 |
| HP0118 |
| HP0121 |
| HP0123 |
| HP0124 |
| HP0125 |
| HP0126 |
| HP0127 |
| HP0129 |
| HP0130 |
| HP0132 |
| HP0133 |
| HP0134 |
| HP0136 |
| HP0137 |
| HP0138 |
| HP0139 |
| HP0140 |
| HP0141 |
| HP0142 |
| HP0143 |
| HP0144 |
| HP0145 |
| HP0146 |
| HP0147 |
| HP0148 |
| HP0149 |
| HP0151 |
| HP0152 |
| HP0153 |
| HP0154 |
| HP0155 |
| HP0156 |
| HP0157 |
| HP0158 |
| HP0159 |
| HP0160 |
| HP0162 |
| HP0163 |
| HP0164 |
| HP0166 |
| HP0167 |
| HP0168 |
| HP0169 |
| HP0170 |
| HP0171 |
| HP0173 |
| HP0174 |
| HP0175 |
| HP0176 |
| HP0177 |
| HP0178 |
| HP0179 |
| HP0180 |
| HP0181 |
| HP0182 |
| HP0183 |
| HP0184 |
| HP0185 |
| HP0189 |
| HP0190 |
| HP0191 |
| HP0192 |
| HP0193 |
| HP0194 |
| HP0195 |
| HP0196 |
| HP0197 |
| HP0198 |
| HP0199 |
| HP0201 |
| HP0202 |
| HP0203 |
| HP0204 |
| HP0207 |
| HP0208 |
| HP0209 |
| HP0210 |
| HP0211 |
| HP0212 |
| HP0213 |
| HP0214 |
| HP0215 |
| HP0216 |
| HP0218 |
| HP0219 |
| HP0220 |
| HP0221 |
| HP0223 |
| HP0224 |
| HP0226 |
| HP0227 |
| HP0228 |
| HP0229 |
| HP0230 |
| HP0231 |
| HP0232 |
| HP0233 |
| HP0234 |
| HP0235 |
| HP0236 |
| HP0237 |
| HP0238 |
| HP0239 |
| HP0240 |
| HP0241 |
| HP0242 |
| HP0243 |
| HP0244 |
| HP0245 |
| HP0246 |
| HP0247 |
| HP0248 |
| HP0249 |
| HP0250 |
| HP0252 |
| HP0254 |
| HP0255 |
| HP0256 |
| HP0257 |
| HP0258 |
| HP0259 |
| HP0260 |
| HP0263 |
| HP0264 |
| HP0265 |
| HP0266 |
| HP0267 |
| HP0268 |
| HP0269 |
| HP0270 |
| HP0271 |
| HP0272 |
| HP0273 |
| HP0274 |
| HP0275 |
| HP0276 |
| HP0277 |
| HP0278 |
| HP0279 |
| HP0280 |
| HP0281 |
| HP0282 |
| HP0283 |
| HP0284 |
| HP0285 |
| HP0286 |
| HP0287 |
| HP0288 |
| HP0290 |
| HP0291 |
| HP0292 |
| HP0293 |
| HP0294 |
| HP0295 |
| HP0296 |
| HP0297 |
| HP0298 |
| HP0299 |
| HP0301 |
| HP0302 |
| HP0303 |
| HP0304 |
| HP0305 |
| HP0306 |
| HP0307 |
| HP0308 |
| HP0309 |
| HP0311 |
| HP0312 |
| HP0313 |
| HP0318 |
| HP0319 |
| HP0321 |
| HP0322 |
| HP0323 |
| HP0324 |
| HP0325 |
| HP0327 |
| HP0328 |
| HP0329 |
| HP0330 |
| HP0331 |
| HP0332 |
| HP0333 |
| HP0334 |
| HP0337 |
| HP0338 |
| HP0347 |
| HP0348 |
| HP0349 |
| HP0350 |
| HP0351 |
| HP0352 |
| HP0353 |
| HP0354 |
| HP0355 |
| HP0357 |
| HP0358 |
| HP0360 |
| HP0361 |
| HP0362 |
| HP0363 |
| HP0364 |
| HP0366 |
| HP0367 |
| HP0369 |
| HP0370 |
| HP0371 |
| HP0372 |
| HP0373 |
| HP0374 |
| HP0375 |
| HP0376 |
| HP0377 |
| HP0378 |
| HP0379 |
| HP0380 |
| HP0381 |
| HP0383 |
| HP0384 |
| HP0385 |
| HP0386 |
| HP0387 |
| HP0388 |
| HP0389 |
| HP0390 |
| HP0391 |
| HP0392 |
| HP0393 |
| HP0394 |
| HP0395 |
| HP0396 |
| HP0397 |
| HP0399 |
| HP0400 |
| HP0401 |
| HP0402 |
| HP0403 |
| HP0404 |
| HP0405 |
| HP0406 |
| HP0407 |
| HP0408 |
| HP0409 |
| HP0410 |
| HP0415 |
| HP0416 |
| HP0417 |
| HP0418 |
| HP0419 |
| HP0420 |
| HP0421 |
| HP0422 |
| HP0459 |
| HP0463 |
| HP0465 |
| HP0466 |
| HP0467 |
| HP0468 |
| HP0469 |
| HP0470 |
| HP0471 |
| HP0472 |
| HP0473 |
| HP0475 |
| HP0476 |
| HP0478 |
| HP0479 |
| HP0480 |
| HP0485 |
| HP0486 |
| HP0487 |
| HP0490 |
| HP0491 |
| HP0492 |
| HP0493 |
| HP0494 |
| HP0495 |
| HP0496 |
| HP0497 |
| HP0498 |
| HP0500 |
| HP0501 |
| HP0506 |
| HP0507 |
| HP0508 |
| HP0509 |
| HP0510 |
| HP0512 |
| HP0514 |
| HP0515 |
| HP0516 |
| HP0517 |
| HP0518 |
| HP0519 |
| HP0549 |
| HP0550 |
| HP0551 |
| HP0553 |
| HP0554 |
| HP0555 |
| HP0557 |
| HP0558 |
| HP0559 |
| HP0561 |
| HP0562 |
| HP0563 |
| HP0564 |
| HP0565 |
| HP0566 |
| HP0567 |
| HP0568 |
| HP0569 |
| HP0570 |
| HP0571 |
| HP0572 |
| HP0573 |
| HP0574 |
| HP0575 |
| HP0576 |
| HP0577 |
| HP0578 |
| HP0579 |
| HP0580 |
| HP0581 |
| HP0583 |
| HP0584 |
| HP0585 |
| HP0586 |
| HP0587 |
| HP0588 |
| HP0589 |
| HP0590 |
| HP0591 |
| HP0594 |
| HP0595 |
| HP0596 |
| HP0597 |
| HP0598 |
| HP0599 |
| HP0601 |
| HP0602 |
| HP0603 |
| HP0604 |
| HP0605 |
| HP0606 |
| HP0607 |
| HP0608 |
| HP0609 |
| HP0614 |
| HP0615 |
| HP0616 |
| HP0617 |
| HP0618 |
| HP0620 |
| HP0621 |
| HP0622 |
| HP0623 |
| HP0624 |
| HP0625 |
| HP0626 |
| HP0627 |
| HP0628 |
| HP0629 |
| HP0630 |
| HP0631 |
| HP0632 |
| HP0633 |
| HP0634 |
| HP0635 |
| HP0637 |
| HP0638 |
| HP0639 |
| HP0640 |
| HP0643 |
| HP0644 |
| HP0645 |
| HP0646 |
| HP0647 |
| HP0648 |
| HP0649 |
| HP0650 |
| HP0652 |
| HP0653 |
| HP0654 |
| HP0655 |
| HP0656 |
| HP0657 |
| HP0658 |
| HP0659 |
| HP0660 |
| HP0661 |
| HP0662 |
| HP0663 |
| HP0664 |
| HP0665 |
| HP0666 |
| HP0671 |
| HP0672 |
| HP0675 |
| HP0676 |
| HP0679 |
| HP0680 |
| HP0683 |
| HP0685 |
| HP0686 |
| HP0687 |
| HP0690 |
| HP0691 |
| HP0692 |
| HP0693 |
| HP0694 |
| HP0696 |
| HP0697 |
| HP0699 |
| HP0700 |
| HP0701 |
| HP0702 |
| HP0703 |
| HP0706 |
| HP0707 |
| HP0708 |
| HP0709 |
| HP0710 |
| HP0711 |
| HP0714 |
| HP0715 |
| HP0716 |
| HP0718 |
| HP0719 |
| HP0720 |
| HP0721 |
| HP0723 |
| HP0724 |
| HP0726 |
| HP0727 |
| HP0728 |
| HP0730 |
| HP0734 |
| HP0735 |
| HP0736 |
| HP0737 |
| HP0738 |
| HP0739 |
| HP0740 |
| HP0741 |
| HP0742 |
| HP0743 |
| HP0745 |
| HP0746 |
| HP0747 |
| HP0748 |
| HP0749 |
| HP0750 |
| HP0751 |
| HP0752 |
| HP0753 |
| HP0754 |
| HP0755 |
| HP0756 |
| HP0757 |
| HP0758 |
| HP0759 |
| HP0760 |
| HP0761 |
| HP0762 |
| HP0763 |
| HP0771 |
| HP0772 |
| HP0773 |
| HP0774 |
| HP0775 |
| HP0776 |
| HP0777 |
| HP0778 |
| HP0779 |
| HP0781 |
| HP0782 |
| HP0783 |
| HP0785 |
| HP0786 |
| HP0787 |
| HP0788 |
| HP0791 |
| HP0792 |
| HP0793 |
| HP0794 |
| HP0795 |
| HP0796 |
| HP0797 |
| HP0798 |
| HP0799 |
| HP0800 |
| HP0802 |
| HP0803 |
| HP0804 |
| HP0805 |
| HP0806 |
| HP0807 |
| HP0808 |
| HP0809 |
| HP0810 |
| HP0811 |
| HP0812 |
| HP0813 |
| HP0814 |
| HP0815 |
| HP0816 |
| HP0817 |
| HP0818 |
| HP0819 |
| HP0820 |
| HP0821 |
| HP0822 |
| HP0823 |
| HP0824 |
| HP0825 |
| HP0826 |
| HP0827 |
| HP0828 |
| HP0829 |
| HP0830 |
| HP0831 |
| HP0832 |
| HP0833 |
| HP0834 |
| HP0835 |
| HP0837 |
| HP0838 |
| HP0839 |
| HP0840 |
| HP0841 |
| HP0842 |
| HP0851 |
| HP0853 |
| HP0854 |
| HP0857 |
| HP0858 |
| HP0859 |
| HP0860 |
| HP0861 |
| HP0862 |
| HP0863 |
| HP0864 |
| HP0865 |
| HP0866 |
| HP0867 |
| HP0868 |
| HP0869 |
| HP0870 |
| HP0871 |
| HP0872 |
| HP0873 |
| HP0874 |
| HP0875 |
| HP0876 |
| HP0877 |
| HP0883 |
| HP0884 |
| HP0885 |
| HP0886 |
| HP0888 |
| HP0889 |
| HP0890 |
| HP0891 |
| HP0898 |
| HP0899 |
| HP0900 |
| HP0902 |
| HP0906 |
| HP0907 |
| HP0908 |
| HP0909 |
| HP0910 |
| HP0911 |
| HP0912 |
| HP0913 |
| HP0918 |
| HP0919 |
| HP0920 |
| HP0921 |
| HP0923 |
| HP0924 |
| HP0925 |
| HP0926 |
| HP0927 |
| HP0928 |
| HP0929 |
| HP0930 |
| HP0931 |
| HP0933 |
| HP0934 |
| HP0935 |
| HP0939 |
| HP0940 |
| HP0941 |
| HP0942 |
| HP0943 |
| HP0944 |
| HP0947 |
| HP0948 |
| HP0949 |
| HP0950 |
| HP0951 |
| HP0952 |
| HP0953 |
| HP0955 |
| HP0956 |
| HP0957 |
| HP0958 |
| HP0959 |
| HP0960 |
| HP0961 |
| HP0969 |
| HP0970 |
| HP0971 |
| HP0972 |
| HP0974 |
| HP0975 |
| HP0976 |
| HP0977 |
| HP0978 |
| HP0979 |
| HP0983 |
| HP1010 |
| HP1011 |
| HP1012 |
| HP1013 |
| HP1014 |
| HP1015 |
| HP1016 |
| HP1017 |
| HP1019 |
| HP1020 |
| HP1021 |
| HP1022 |
| HP1024 |
| HP1025 |
| HP1026 |
| HP1027 |
| HP1028 |
| HP1029 |
| HP1030 |
| HP1031 |
| HP1032 |
| HP1034 |
| HP1035 |
| HP1036 |
| HP1037 |
| HP1038 |
| HP1039 |
| HP1040 |
| HP1041 |
| HP1042 |
| HP1043 |
| HP1044 |
| HP1046 |
| HP1047 |
| HP1048 |
| HP1049 |
| HP1050 |
| HP1051 |
| HP1052 |
| HP1053 |
| HP1054 |
| HP1055 |
| HP1056 |
| HP1058 |
| HP1059 |
| HP1060 |
| HP1061 |
| HP1062 |
| HP1063 |
| HP1064 |
| HP1065 |
| HP1066 |
| HP1067 |
| HP1068 |
| HP1069 |
| HP1071 |
| HP1072 |
| HP1073 |
| HP1075 |
| HP1076 |
| HP1077 |
| HP1080 |
| HP1081 |
| HP1082 |
| HP1083 |
| HP1084 |
| HP1085 |
| HP1086 |
| HP1087 |
| HP1088 |
| HP1089 |
| HP1090 |
| HP1091 |
| HP1092 |
| HP1098 |
| HP1099 |
| HP1100 |
| HP1101 |
| HP1102 |
| HP1103 |
| HP1104 |
| HP1105 |
| HP1106 |
| HP1107 |
| HP1108 |
| HP1109 |
| HP1110 |
| HP1111 |
| HP1112 |
| HP1113 |
| HP1114 |
| HP1117 |
| HP1118 |
| HP1119 |
| HP1120 |
| HP1121 |
| HP1122 |
| HP1123 |
| HP1124 |
| HP1125 |
| HP1126 |
| HP1127 |
| HP1129 |
| HP1130 |
| HP1131 |
| HP1132 |
| HP1133 |
| HP1134 |
| HP1135 |
| HP1136 |
| HP1137 |
| HP1138 |
| HP1139 |
| HP1140 |
| HP1141 |
| HP1143 |
| HP1147 |
| HP1148 |
| HP1149 |
| HP1150 |
| HP1151 |
| HP1152 |
| HP1153 |
| HP1154 |
| HP1155 |
| HP1156 |
| HP1158 |
| HP1159 |
| HP1160 |
| HP1161 |
| HP1162 |
| HP1163 |
| HP1164 |
| HP1166 |
| HP1167 |
| HP1168 |
| HP1169 |
| HP1170 |
| HP1171 |
| HP1172 |
| HP1173 |
| HP1174 |
| HP1175 |
| HP1177 |
| HP1178 |
| HP1179 |
| HP1180 |
| HP1181 |
| HP1182 |
| HP1183 |
| HP1184 |
| HP1185 |
| HP1186 |
| HP1189 |
| HP1190 |
| HP1191 |
| HP1195 |
| HP1196 |
| HP1197 |
| HP1198 |
| HP1199 |
| HP1200 |
| HP1201 |
| HP1202 |
| HP1203 |
| HP1203a |
| HP1205 |
| HP1206 |
| HP1207 |
| HP1208 |
| HP1210 |
| HP1212 |
| HP1213 |
| HP1214 |
| HP1215 |
| HP1216 |
| HP1217 |
| HP1218 |
| HP1220 |
| HP1221 |
| HP1222 |
| HP1223 |
| HP1224 |
| HP1225 |
| HP1226 |
| HP1227 |
| HP1228 |
| HP1229 |
| HP1230 |
| HP1231 |
| HP1232 |
| HP1233 |
| HP1234 |
| HP1235 |
| HP1236 |
| HP1237 |
| HP1238 |
| HP1240 |
| HP1241 |
| HP1242 |
| HP1243 |
| HP1244 |
| HP1245 |
| HP1246 |
| HP1247 |
| HP1248 |
| HP1249 |
| HP1250 |
| HP1253 |
| HP1254 |
| HP1255 |
| HP1256 |
| HP1257 |
| HP1258 |
| HP1259 |
| HP1260 |
| HP1261 |
| HP1262 |
| HP1263 |
| HP1264 |
| HP1265 |
| HP1266 |
| HP1267 |
| HP1268 |
| HP1269 |
| HP1270 |
| HP1271 |
| HP1273 |
| HP1274 |
| HP1275 |
| HP1277 |
| HP1278 |
| HP1279 |
| HP1280 |
| HP1281 |
| HP1282 |
| HP1284 |
| HP1285 |
| HP1289 |
| HP1290 |
| HP1291 |
| HP1292 |
| HP1293 |
| HP1294 |
| HP1295 |
| HP1296 |
| HP1297 |
| HP1298 |
| HP1299 |
| HP1300 |
| HP1301 |
| HP1302 |
| HP1303 |
| HP1304 |
| HP1305 |
| HP1307 |
| HP1308 |
| HP1309 |
| HP1310 |
| HP1311 |
| HP1312 |
| HP1313 |
| HP1314 |
| HP1315 |
| HP1316 |
| HP1317 |
| HP1318 |
| HP1319 |
| HP1320 |
| HP1321 |
| HP1322 |
| HP1323 |
| HP1325 |
| HP1326 |
| HP1327 |
| HP1328 |
| HP1329 |
| HP1330 |
| HP1331 |
| HP1332 |
| HP1333 |
| HP1335 |
| HP1336 |
| HP1337 |
| HP1338 |
| HP1339 |
| HP1340 |
| HP1343 |
| HP1344 |
| HP1345 |
| HP1346 |
| HP1347 |
| HP1348 |
| HP1349 |
| HP1350 |
| HP1355 |
| HP1356 |
| HP1357 |
| HP1358 |
| HP1359 |
| HP1360 |
| HP1361 |
| HP1362 |
| HP1363 |
| HP1364 |
| HP1365 |
| HP1372 |
| HP1373 |
| HP1374 |
| HP1375 |
| HP1376 |
| HP1377 |
| HP1378 |
| HP1379 |
| HP1380 |
| HP1384 |
| HP1385 |
| HP1386 |
| HP1387 |
| HP1391 |
| HP1392 |
| HP1393 |
| HP1394 |
| HP1395 |
| HP1398 |
| HP1399 |
| HP1401 |
| HP1406 |
| HP1407 |
| HP1413 |
| HP1414 |
| HP1415 |
| HP1416 |
| HP1418 |
| HP1419 |
| HP1420 |
| HP1421 |
| HP1422 |
| HP1423 |
| HP1424 |
| HP1428 |
| HP1429 |
| HP1430 |
| HP1431 |
| HP1434 |
| HP1435 |
| HP1436 |
| HP1440 |
| HP1441 |
| HP1442 |
| HP1443 |
| HP1444 |
| HP1445 |
| HP1446 |
| HP1447 |
| HP1449 |
| HP1450 |
| HP1451 |
| HP1452 |
| HP1453 |
| HP1454 |
| HP1455 |
| HP1456 |
| HP1457 |
| HP1458 |
| HP1459 |
| HP1460 |
| HP1461 |
| HP1462 |
| HP1463 |
| HP1464 |
| HP1465 |
| HP1466 |
| HP1467 |
| HP1468 |
| HP1469 |
| HP1470 |
| HP1473 |
| HP1474 |
| HP1475 |
| HP1476 |
| HP1477 |
| HP1478 |
| HP1479 |
| HP1480 |
| HP1481 |
| HP1482 |
| HP1483 |
| HP1484 |
| HP1485 |
| HP1486 |
| HP1487 |
| HP1488 |
| HP1489 |
| HP1490 |
| HP1491 |
| HP1492 |
| HP1493 |
| HP1494 |
| HP1495 |
| HP1496 |
| HP1497 |
| HP1498 |
| HP1502 |
| HP1504 |
| HP1505 |
| HP1506 |
| HP1507 |
| HP1508 |
| HP1509 |
| HP1510 |
| HP1511 |
| HP1513 |
| HP1514 |
| HP1517 |
| HP1523 |
| HP1524 |
| HP1525 |
| HP1526 |
| HP1527 |
| HP1529 |
| HP1530 |
| HP1531 |
| HP1532 |
| HP1533 |
| HP1538 |
| HP1539 |
| HP1540 |
| HP1541 |
| HP1542 |
| HP1543 |
| HP1544 |
| HP1545 |
| HP1546 |
| HP1547 |
| HP1548 |
| HP1549 |
| HP1550 |
| HP1551 |
| HP1552 |
| HP1553 |
| HP1554 |
| HP1555 |
| HP1556 |
| HP1557 |
| HP1558 |
| HP1559 |
| HP1560 |
| HP1562 |
| HP1563 |
| HP1564 |
| HP1565 |
| HP1566 |
| HP1567 |
| HP1568 |
| HP1569 |
| HP1570 |
| HP1571 |
| HP1572 |
| HP1573 |
| HP1574 |
| HP1575 |
| HP1576 |
| HP1577 |
| HP1579 |
| HP1580 |
| HP1581 |
| HP1582 |
| HP1583 |
| HP1584 |
| HP1585 |
| HP1588 |
| JHP0533 |
| HELPY_0329 |
| HELPY_0330 |
