# Supplementary material for: New Implications on Genomic Adaptation Derived from the Helicobacter pylori Genome Comparison
Source: PLoS One. 2011 Feb 28;6(2):e17300. doi: 10.1371/journal.pone.0017300 (PMC3046158; doi:10.1371/journal.pone.0017300)
Supplement: Table S3 — The mutations responsible for protein premature in strain B38. (DOCX) [file pone.0017300.s005.docx]

Table S3. The mutations responsible for protein premature in strain B38

| **Pseudogenes** | **Homont indel^1^** | **Heteront indel^2^** | **Conversion to stop codon** | **Tandem rep indel^3^** | **Direct rep indel^4^** | **Homologous recom.^5^** | **Transposon** | **Proteins** |
| --- | --- | --- | --- | --- | --- | --- | --- | --- |
| HELPY_0046 |  |  |  |  | TG, |  |  | type II cytosine methyltransferase |
| HELPY_0047 |  |  |  |  | 181bp del^6^ |  |  |  |
| HELPY_0053 |  |  |  |  | TGAAAAAATC, | |  | conserved hypothetical protein |
| HELPY_0054 |  |  |  |  | 32 bp del |  |  |  |
| HELPY_0060 | 5C/6C^8^ |  |  |  |  |  |  | putative ATP-binding protein |
| HELPY_0061 | 9A/8A |  |  |  |  |  |  |  |
| HELPY_0062 |  |  |  |  |  |  |  |  |
| HELPY_0088 | 4A/5A |  |  |  |  |  |  | type II restriction enzyme MboI |
| HELPY_0089 |  |  |  |  |  |  |  |  |
| HELPY_0107 | 8A/7A |  |  |  |  |  |  | conserved hypothetical protein |
| HELPY_0108 |  |  |  |  |  |  |  |  |
| HELPY_0118 | 1A/3A |  |  |  |  |  |  | conserved hypothetical protein |
| HELPY_0119 |  |  |  |  |  |  |  |  |
| HELPY_0190 |  |  | TAA/GAA^9^ |  |  |  |  | conserved hypothetical protein |
| HELPY_0191 |  |  |  |  |  |  |  |  |
| HELPY_0208 |  |  |  |  |  | GGTGA/ |  | conserved hypothetical protein |
| HELPY_0209 |  |  |  |  |  | GACAA |  |  |
| HELPY_0220 | 11C/12C |  |  |  |  |  |  | putative beta-1,4-N- |
| HELPY_0221 |  |  |  |  |  |  |  | acetylgalactosamyltransferase |
| HELPY_0226 | 6T/7T |  |  |  |  |  |  | conserved hypothetical protein |
| HELPY_0227 |  |  |  |  |  |  |  |  |
| HELPY_0256 | 6C/7C |  |  |  |  |  |  | ABC-type transport sytem, permease, |
| HELPY_0257 |  |  |  |  |  |  |  | putative membrane preotein |
| HELPY_0267 |  |  |  | ATTA1 |  |  |  | non-functional type II restriction |
| HELPY_0268 |  |  |  | Ins^7^ |  |  |  | endonuclease |
| HELPY_0342 | 2C/3C |  |  |  |  |  |  | lysozyme |
| HELPY_0343 | 6A/7A |  |  |  |  |  |  |  |
| HELPY_0344 |  |  |  |  |  |  |  |  |
| HELPY_0347 |  |  | TAA/CAA |  |  |  |  | conserved hypothetical protein |
| HELPY_0348 |  |  |  |  |  |  |  |  |
| HELPY_0349 |  |  |  |  |  |  |  |  |
| HELPY_0446 | 11C/12C |  |  |  |  |  |  | type I restriction enzyme R protein |
| HELPY_0447 |  |  |  |  |  |  |  |  |
| HELPY_0497 |  |  |  | 13GA/5GA | |  |  | alginate O-acetylation protein AlgI |
| HELPY_0498 |  |  |  |  |  |  |  |  |
| HELPY_0502 |  | TGT/TT | |  |  |  |  | conserved hypothetical protein |
| HELPY_0503 |  |  |  |  |  |  |  |  |
| HELPY_0505 | 7C/6C |  |  |  |  |  |  | putative type I R-M modification protein |
| HELPY_0506 |  |  |  |  |  |  |  |  |
| HELPY_0508 |  |  |  | AAAT,ins |  |  |  | putative type I R-M restriction enzyme R |
| HELPY_0509 |  |  |  |  |  |  |  | protein |
| HELPY_0550 | 8A/7A |  | TAA/CAA |  |  |  |  |  |
| HELPY_0551 |  |  |  |  |  |  |  | iron(III) dicitrate transport protein FecA |
| HELPY_0552 |  |  |  |  |  |  |  |  |
| HELPY_0597 |  |  |  | 20bpX1, ins | |  |  | conserved hypothetical protein |
| HELPY_0598 |  |  |  |  |  |  |  |  |
| HELPY_0621 |  |  |  | 8GA/6GA |  |  |  | conserved hypothetical protein |
| HELPY_0622 | 5G/4G |  |  |  |  |  |  |  |
| HELPY_0623 |  |  |  |  |  |  |  |  |
| HELPY_0634 | A8/9A |  |  |  | GAGCGATT, |  |  | conserved hypothetical protein |
| HELPY_0635 |  |  |  |  | 22bp del. |  |  |  |
| HELPY_0653 |  |  |  |  |  | 22bp del |  | conserved hypothetical protein |
| HELPY_0654 |  |  |  |  |  |  |  |  |
| HELPY_0663 |  |  |  |  |  | 52bp del |  | conserved hypothetical protein |
| HELPY_0664 |  |  |  |  |  |  |  |  |
| HELPY_0675 |  |  |  | 106 bp X2, ins | |  |  | hydantoin utilization protein A HyuA |
| HELPY_0676 |  |  |  |  |  |  |  |  |
| HELPY_0677 |  |  |  |  |  |  |  |  |
| HELPY_0699 |  |  |  |  |  |  | transposon | type I restriction-modification polypeptide |
| HELPY_0704 |  |  |  |  |  |  |  |  |
| HELPY_0742 |  |  |  | 2AT/AT |  |  |  | conserved hypothetical protein |
| HELPY_0743 |  |  |  |  |  |  |  |  |
| HELPY_0752 | 13C/14C |  |  |  |  |  |  | putative lipopolysaccharide biosynthesis |
| HELPY_0753 |  |  |  |  |  |  |  | protein |
| HELPY_0759 |  |  |  |  |  | CCACA, del | | putative ABC-type transport system，ATP |
| HELPY_0760 |  |  |  |  |  |  |  | binding protein , transmembrane protein |
| HELPY_0761 | 2A/A |  |  |  |  |  |  | putative ABC-type transport system， |
| HELPY_0762 |  |  |  |  |  |  |  | permease |
| HELPY_0834 |  |  |  |  |  |  | transposon | putative ABC transporter, permease |
| HELPY_0839 |  |  |  |  |  |  |  |  |
| HELPY_0848 |  |  |  |  | CCA, |  |  | conserved hypothetical protein |
| HELPY_0849 |  |  |  |  | 5 bp del |  |  |  |
| HELPY_0853 | 8C/9C |  |  |  |  |  |  | outer membrane phospholipase A1 |
| HELPY_0854 |  |  |  |  |  |  |  | precursor PldA |
| HELPY_0866 | 3T/4T,A/2A | |  |  |  |  |  | conserved prophage protein |
| HELPY_0867 |  |  |  |  |  |  |  |  |
| HELPY_0887 |  |  |  |  |  | ACGCC ins | | putative acetate kinase AckA |
| HELPY_0888 |  |  |  |  |  |  |  |  |
| HELPY_0920 |  |  |  |  | GG, |  |  | putative proline/betaine transporter, |
| HELPY_0921 |  |  |  |  | 4bp del |  |  | transmembrane |
| HELPY_0922 |  | AAT/ATAT | |  |  |  |  | conserved hypothetical protein |
| HELPY_0923 |  |  |  |  |  |  |  |  |
| HELPY_0950 | 6A/7A | G/GC |  |  |  |  |  | conserved hypothetical protein |
| HELPY_0951 |  |  |  |  |  |  |  |  |
| HELPY_0952 |  |  |  |  |  |  |  |  |
| HELPY_0953 |  |  | TAA/CTT |  |  |  |  | conserved hypothetical protein |
| HELPY_0954 |  |  |  |  |  |  |  |  |
| HELPY_0978 | A8/7A |  |  |  |  |  |  | conserved hypothetical protein |
| HELPY_0979 |  |  |  |  |  |  |  |  |
| HELPY_0980 |  |  |  |  | AT or ATAAA, |  |  | conserved hypothetical protein |
| HELPY_0981 |  |  |  |  | del 11 bp |  |  |  |
| HELPY_0982 | 2A/3A |  |  |  |  |  |  | conserved hypothetical protein |
| HELPY_0983 | A5/6A |  |  |  |  |  |  | conserved hypothetical protein |
| HELPY_0984 |  |  |  |  |  |  |  |  |
| HELPY_0985 | C5/4C |  |  |  |  |  |  | conserved hypothetical protein |
| HELPY_0986 |  |  |  |  |  |  |  |  |
| HELPY_0991 | 6T/5T |  |  |  |  |  |  | putative phosphatase |
| HELPY_1004 |  |  |  |  |  | 41 bp ins |  | conserved hypothetical protein |
| HELPY_1005 |  |  |  |  |  |  |  |  |
| HELPY_1183 | 5G/6G |  |  |  |  |  |  | type II restriction endonuclease IceA1 |
| HELPY_1184 |  |  |  |  |  | ATTA/ACA | |  |
| HELPY_1185 |  |  |  |  |  |  |  |  |
| HELPY_1218 |  |  |  |  |  |  |  | adhesin BabA |
| HELPY_1219 | 2A/A |  |  |  |  |  |  |  |
| HELPY_1330 |  |  |  |  |  |  | transposon | conserved hypothetical protein |
| HELPY_1335 |  |  |  |  |  |  |  |  |
| HELPY_1342 | 12C/14C |  |  |  |  |  |  | putative adenine specific DNA |
| HELPY_1343 | 15C/13C |  |  |  |  |  |  |  |
| HELPY_1344 |  |  |  |  |  |  |  | methyltransferase |
| HELPY_1356 | G13/15G |  |  |  |  |  |  | putative type III restriction enzyme M |
| HELPY_1357 |  |  |  |  |  |  |  | protein |
| HELPY_1378 | 2T/T |  |  |  |  |  |  | conserved hypothetical protein |
| HELPY_1379 |  |  |  |  |  |  |  |  |
| HELPY_1385 |  |  |  | 9GA/10GA | |  |  | conserved hypothetical membrane protein |
| HELPY_1386 |  |  |  |  |  |  |  |  |
| HELPY_1394 | 6T/7T |  |  |  |  |  |  | conserved hypothetical protein |
| HELPY_1395 |  |  |  |  |  |  |  |  |
| HELPY_1443 | 10G/16G |  |  |  |  |  |  | putative type IIS restriction enzyme |
| HELPY_1444 |  |  |  |  |  |  |  |  |
| HELPY_1445 |  |  |  |  |  |  |  |  |
| HELPY_1488 | 3T/4T |  |  |  |  |  |  | conserved hypothetical protein |
| HELPY_1489 |  |  |  |  |  |  |  |  |
| HELPY_1493 | 11G/12G |  |  |  |  |  |  | putative type III R-M system modification |
| HELPY_1494 |  |  |  |  |  |  |  | enzyme |
| HELPY_1528 |  |  |  |  |  |  | transposon | putative Hac prophage II protein |
| HELPY_1533 |  |  |  |  |  |  |  |  |
| Total | 36 | 3 | 4 | 8 | 6 | 7 | 4 |  |

Note:

1. Homonucleotide insertion and deletion

2. Heteronucleotide insertion and deletion

3. Tandem repeat insertion and deletion

4. Direct repeat insertion and deletion

5. Homologous recombination

6. Deletion

7. Insertion

8. 5C/6C, represents that the normal (inframe) sequence, 6 homonucleotides “C” was replaced by 5 “C”.

9. TAA/GAA means that the normal (inframe) sequence, GAA was replaced by TAA.
