# Supplementary material for: New Implications on Genomic Adaptation Derived from the Helicobacter pylori Genome Comparison
Source: PLoS One. 2011 Feb 28;6(2):e17300. doi: 10.1371/journal.pone.0017300 (PMC3046158; doi:10.1371/journal.pone.0017300)
Supplement: Table S4 — The mutations responsible for protein premature in other genomes. (DOCX) [file pone.0017300.s006.docx]

**Table S4 The mutations responsible for protein premature in other genomes**

| **Pseudogenes** | **Homont indel**^1^ | **Heteront indel**^2^ | **Conversion stop codon** | **Tandem rep indel**^3^ | **Direct rep indel**^4^ | **Homologous recom**^5^ | **Transposon** | **Gene** | |
| --- | --- | --- | --- | --- | --- | --- | --- | --- | --- |
| HP0066 |  |  |  | AAAGCATTTG | | |  | |  |
|  |  |  |  | (HPKB_0075) | | |  |  |  |
| HP0069 | 2T/3T^6^ | |  |  |  |  |  | | urease accessory protein (ureF) |
|  | (HPG27_64)^7^ | |  |  |  |  |  |  |  |
| HP0091 | T/2T | ATA/AA | |  |  |  |  | | type II restriction enzyme R protein  (hsdR) |
|  | (jhp_0084) | (jhp_0084) | |  |  |  |  |  |  |
|  | 4A/5A | TCA/TA | |  |  |  |  | |  |
|  | (HELPY_0088_0089) | (jhp_0084) | |  |  |  |  |  |  |
|  | 5T/4T | |  |  |  |  |  | |  |
|  | (HPSH_00450) | |  |  |  |  |  |  |  |
|  | C/2C | | |  |  |  |  | |  |
|  | (HPP12_0094_0095) | | |  |  |  |  |  |  |
| HP0150 | 4T/5 T | |  |  |  |  |  | | hypothetical protein |
|  | (HPG27_0137) | |  |  |  |  |  |  |  |
| HP0209 |  |  |  | 11AG/10AG (HPG27_190, HP_0209) | 19 bp deleted by | | | | Outer membrane protein (hofA),  phase-variable gene |
|  |  |  |  |  | TAAAG repeat | | | |  |
|  |  |  |  |  | (HPP12_0206_0207) | | | |  |
| HP0251 | 6C/7C | | |  |  |  |  | | oligopeptide ABC transporter,  permease protein (oppC) |
|  | (HELPY_0256_0257) | | |  |  |  |  |  |  |
|  | 2A/A | |  |  |  |  |  | |  |
|  | (HPP12_0251) | |  |  |  |  |  |  |  |
| HP0320 | 7A/6A | |  |  |  |  |  | | Sec-independent protein translocase  protein tatA/E homolog |
|  | (HPG27_0301) | |  |  |  |  |  |  |  |
| HP0368 | 6A/5A | |  |  |  |  |  | | type II R-M system restriction  endonuclease |
|  | (HPKB_1015) | |  |  |  |  |  |  |  |
|  | 6T/4 T | |  |  |  |  |  | |  |
|  | (HPAG1_1025) | |  |  |  |  |  |  |  |
|  | 7A/8A | |  |  |  |  |  | |  |
|  | (HP_0368) | |  |  |  |  |  |  |  |
| HP0382 | 8T/6 T | |  |  |  |  |  | | putative metalloprotease;  putative membrane protein |
|  | (HPSH_05510) | |  |  |  |  |  |  |  |
| HP0398 | 8A/9A | |  |  |  |  |  | | hypothetical protein |
|  | (HPSH_05415) | |  |  |  |  |  |  |  |
| HP0473 | 6T/7 T | | TAA/CAA |  |  |  |  | | molybdenum ABC transporter ModA |
|  | (HPKB_0455) | |  |  |  |  |  |  |  |
| HP0499 | 9G/8 G  (KHP_0817) | |  |  |  |  |  | | phospholipase A1 precursor  (DR-phospholipase A),  Phase-variable gene |
|  |  | |  |  |  |  |  |  |  |
|  | 7G/8G | |  |  |  |  |  | |  |
|  | (HPG27_459) | |  |  |  |  |  |  |  |
|  | 9G/8 G | | |  |  |  |  | |  |
|  | (HELPY_0853_0854) | | |  |  |  |  |  |  |
| HP0552 | 5A/6A and 6A/7 A | | |  |  |  |  | | tetrapyrrole methylase family protein |
|  | (HPG27_512) | | |  |  |  |  |  |  |
| HP0600 | 8A/7 A | |  |  |  |  |  | | multidrug resistance protein (spaB) |
|  | (HPG27_560) | |  |  |  |  |  |  |  |
|  |  |  |  |  |  | 5 bp deletion, | | |  |
|  |  |  |  |  |  | HPKB_0742_0743 | | |  |
| HP0651 | 12C/13C | | |  |  |  |  | | Alpha1,3-fucosyltransferase (futA),  Phase-variable gene |
|  | (HPAG1_0636) | | |  |  |  |  |  |  |
| HP0688_0689 | 4G/5G |  |  |  |  |  |  | | Nucleic acid binding protein |
| HP0705 | 7T/8T | |  |  |  |  |  | | excinuclease ABC subunit A (uvrA) |
|  | (HPG27_0662) | |  |  |  |  |  |  |  |
| HP0717 | 4T/3 T | |  |  |  |  |  | | DNA polymerase III subunits gamma  and tau |
|  | (HPG27_0673) | |  |  |  |  |  |  |  |
| HP0729 | 6A/7 A | | | |  |  |  | | ATP/GTP binding protein |
|  | (HPP12_0737, | | | |  |  |  |  |  |
|  | HPP12_0738) | | | |  |  |  |  |  |
| HP0770 | 7T/8T | |  |  |  |  |  | | flagellar biosynthesis protein (flhB) |
|  | (HPSH_02960) | |  |  |  |  |  |  |  |
| HP0780 | 7A/8A | |  |  |  |  |  | | hypothetical protein |
|  | (HPSH_02910) | |  |  |  |  |  |  |  |
| HP0878 or HP0879 | 3G/2 G | |  |  |  |  |  | | hypothetical protein |
|  | (HP_0878) | |  |  |  |  |  |  |  |
| HP0914 | 3T/2 T | |  |  |  |  |  | | putative outer membrane protein |
|  | (HPSH_04820) | |  |  |  |  |  |  |  |
| HP0916_0915 | 4G/3 G | |  |  |  |  |  | | iron-regulated outer membrane  protein (frpB) |
|  | (HPKB_0883 ) | |  |  |  |  |  |  |  |
|  | G/2G | |  |  |  |  |  | | iron-regulated outer membrane  protein (frpB) |
|  | (HP0916_0915) | |  |  |  |  |  |  |  |
| HP0932_0931 | 3C/4 C | | |  |  |  |  | | hypothetical protein |
|  | (HP0931_0932) | | |  |  |  |  |  |  |
| HP0946 | 7T/8T | |  |  |  |  |  | | integral membrane protein |
|  | (HPSH_04980) | |  |  |  |  |  |  |  |
| HP1023 | 6G/7G | |  |  |  |  |  | | hypothetical protein |
|  | (HPG27_405) | |  |  |  |  |  |  |  |
| HP1057 | 5G/6 G | |  |  |  |  |  | | hypothetical protein |
|  | (HPG27_371) | |  |  |  |  |  |  |  |
| HP1070 | T8/9T | | |  |  |  |  | | hypothetical protein |
|  | (No annotated in Shi470) | | |  |  |  |  |  |  |
| HP1204 | 4A/5A | | |  |  |  |  | | 50S ribosomal protein L33 (rpmG) |
|  | (No annotated in P12) | | |  |  |  |  |  |  |
| HP1251 |  |  | TAG/TGG | | |  |  | | oligopeptide ABC transporter,  permease protein (oppB) |
|  |  |  | (HPSH_06480) | | |  |  |  |  |
| HP1259 | 3A/2A | |  |  |  |  |  | | NAD-dependent deacetylase |
|  | (HP_1259) | |  |  |  |  |  |  |  |
| HP1272 | 3A/2A, 3C/2C | CAT/CT |  |  |  |  |  | | NADH dehydrogenase subunit M |
|  | (HPG27_1217) | (HPG27_1217) | |  |  |  |  | |  |
| HP1286 | 2G/G | |  |  |  |  |  | | conserved hypothetical secreted protein |
|  | (HPP12_1252) | |  |  |  |  |  |  |  |
| HP1287 | 4T/3T, 7G/6G, 4G/3G | | | |  |  |  | | transcriptional regulator (tenA) |
|  | (HPP12_1253) | | | |  |  |  |  |  |
| HP1403 | 2T/3T | |  |  |  |  |  | | type I restriction enzyme M protein  (hsdM) |
|  | (HPAG1_1464) | |  |  |  |  |  |  |  |
| HP1448 | 7T/8T | |  |  |  |  |  | | ribonuclease P, protein component  (rnpA) |
|  | (HPG27_1369) | |  |  |  |  |  |  |  |
| HP1501 | 2T/T | |  |  |  |  |  | | Outer membrane protein HorK |
|  | (HPSH_07695) | |  |  |  |  |  |  |  |
| HP1503 | 5T/6T | |  |  |  |  |  | | cation-transporting ATPase,  P-type (copA) |
|  | (HPG27_1427) | |  |  |  |  |  |  |  |
| HP1512 | 9G/8G | |  |  |  |  |  | | iron-regulated outer membrane  protein (frpB) |
|  | (HPSH_07760) | |  |  |  |  |  |  |  |
| **Total** | 50 | 3 | 2 | 3 | 1 | 3 | 0 | |  |

Note:

1. Homonucleotide insertion and deletion

2. Heteronucleotide insertion ans deletion

3. Tandem repeat insertion and deletion

4. Direct repeat insertion and deletion

5. Homologous recombination

6. 2T/3T, represents that the normal (inframe) sequence, 3 homonucleotides “T” was replaced by 2 “T”. Others can be extrapolated in similar way.

7. The gene that contains this premature mutation.
